# Compositional complementarity between genomic RNA and coat proteins in positive-sense single-stranded RNA viruses

**DOI:** 10.1093/nar/gkac202

**Published:** 2022-03-31

**Authors:** Marlene Adlhart, Florian Poetsch, Mario Hlevnjak, Megan Hoogmoed, Anton A Polyansky, Bojan Zagrovic

**Affiliations:** Department of Structural and Computational Biology, Max Perutz Labs, University of Vienna, Campus Vienna Biocenter 5, A-1030, Vienna, Austria; Institute for Physiology and Pathophysiology, Center for Medical Research, Johannes Kepler University of Linz, Huemerstraße 3-5, 4020 Linz, Austria; Division of Molecular Genetics, German Cancer Research Center (DKFZ), Im Neuenheimer Feld 580, 69120 Heidelberg, Germany; Department of Structural and Computational Biology, Max Perutz Labs, University of Vienna, Campus Vienna Biocenter 5, A-1030, Vienna, Austria; Department of Structural and Computational Biology, Max Perutz Labs, University of Vienna, Campus Vienna Biocenter 5, A-1030, Vienna, Austria; Department of Structural and Computational Biology, Max Perutz Labs, University of Vienna, Campus Vienna Biocenter 5, A-1030, Vienna, Austria

## Abstract

During packaging in positive-sense single-stranded RNA (+ssRNA) viruses, coat proteins (CPs) interact directly with multiple regions in genomic RNA (gRNA), but the underlying physicochemical principles remain unclear. Here we analyze the high-resolution cryo-EM structure of bacteriophage MS2 and show that the gRNA/CP binding sites, including the known packaging signal, overlap significantly with regions where gRNA nucleobase-density profiles match the corresponding CP nucleobase-affinity profiles. Moreover, we show that the MS2 packaging signal corresponds to the global minimum in gRNA/CP interaction energy in the unstructured state as derived using a linearly additive model and knowledge-based nucleobase/amino-acid affinities. Motivated by this, we predict gRNA/CP interaction sites for a comprehensive set of 1082 +ssRNA viruses. We validate our predictions by comparing them with site-resolved information on gRNA/CP interactions derived in SELEX and CLIP experiments for 10 different viruses. Finally, we show that in experimentally studied systems CPs frequently interact with autologous coding regions in gRNA, in agreement with both predicted interaction energies and a recent proposal that proteins in general tend to interact with own mRNAs, if unstructured. Our results define a self-consistent framework for understanding packaging in +ssRNA viruses and implicate interactions between unstructured gRNA and CPs in the process.

## INTRODUCTION

Understanding how viruses package their genomes is a question of both fundamental and practical importance (1–7). While in most DNA viruses the genome is inserted into a pre-formed capsid by motor proteins (2,8,9), the capsids of single-stranded RNA (ssRNA) viruses typically assemble around the genomic RNA (gRNA) in a highly cooperative, spontaneous process (10–12). This gives rise to two principal challenges. First, it is not clear how viral gRNA and coat proteins (CPs) selectively recognize each other against the background of other competitive interactions in the cell. Second, the mechanism of forming the mature viral particle involves a complex interplay between gRNA and CP folding and capsid assembly, but its details remain unclear (13,14). A paradigm that has emerged over the years is that CPs directly recognize and bind in a hierarchical fashion to multiple regions in gRNA, including packaging signals (PSs), short stretches of gRNA where packaging is initiated (4,7,15–19). The physicochemical determinants of the recognition between CPs and target gRNA are, however, incompletely understood. Related to this, identification and prediction of gRNA/CP interaction sites, including PSs, remains an important open challenge. Finally, it is not clear how gRNA/CP binding relates to other factors which also contribute to packaging selectivity in some viruses, including active RNA replication, translation and formation of viral factories (4).

**Figure 1.**
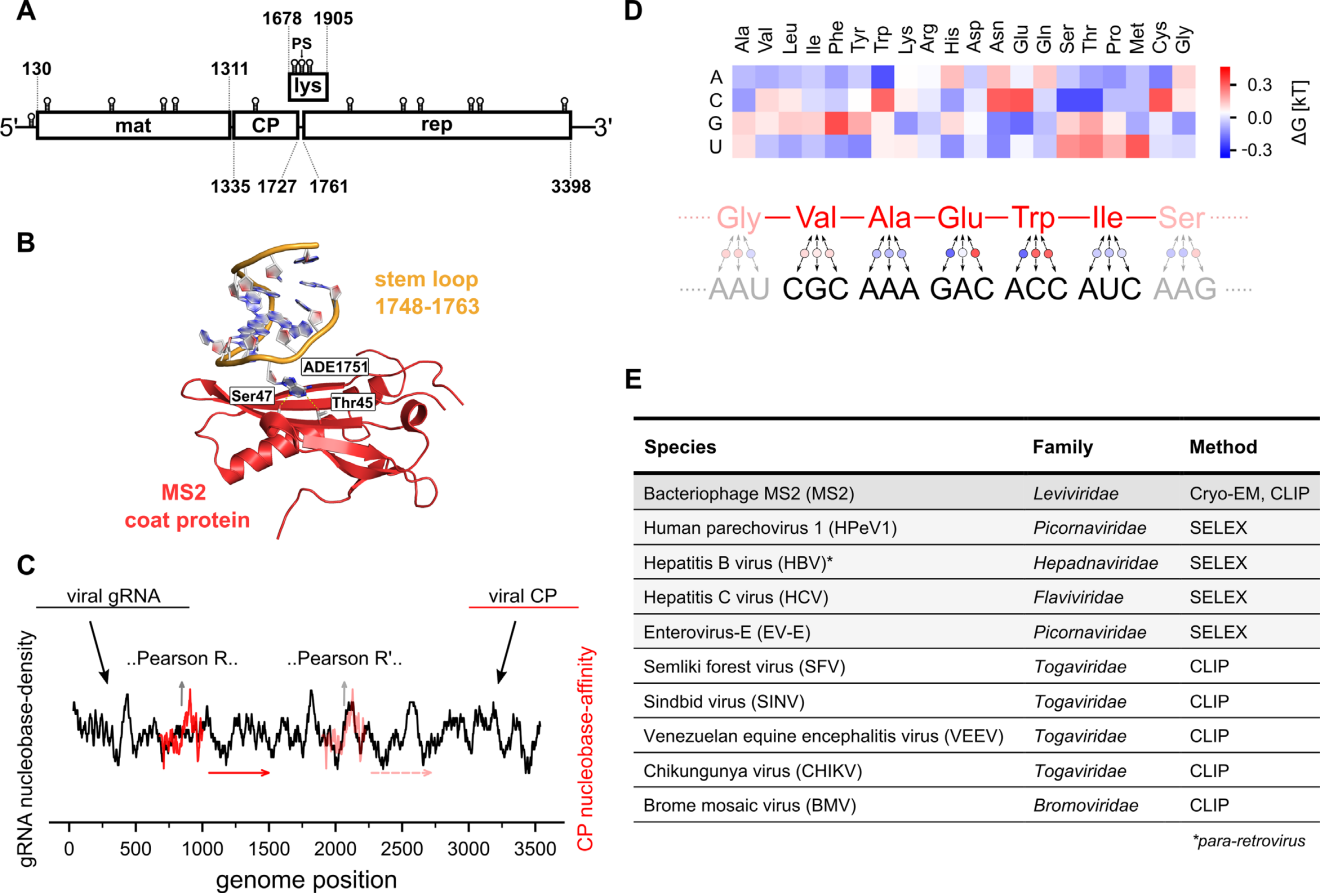
Assessing the relationship between gRNA and CP sequences in +ssRNA viruses. (**A**) MS2 genome organization. Locations of the 15 RNA stem-loops which interact with CP dimers in the MS2 cryo-EM structure are depicted schematically (23). (**B**) High-resolution view of the binding interface between MS2 PS and CP, with key interacting residues labeled (23). (**C**) Sequence profile comparison: at every gRNA position, the matching between the CP nucleobase-affinity profile and the corresponding viral gRNA nucleobase-density profile is quantified via Pearson R. A strong matching between profiles may indicate regions of preferred interaction. (**D**) Prediction of gRNA/CP interaction energy in the unstructured state: the relative interaction energy between a given gRNA fragment and CP is calculated as the sum of the affinities of all individual knowledge-based nucleobase/amino-acid interactions (top), with each amino acid assumed to interact with three consecutive nucleobases after alignment (bottom). (**E**) Analyzed viral species with available data on the interaction between the complete viral gRNA and CP, together with the method used. Note that HBV is a DNA virus, but was included in the analysis since its pre-genomic RNA (+ssRNA) is initially packaged in the capsid (30).

A powerful system for studying packaging in positive-sense ssRNA (+ssRNA) viruses is the bacteriophage MS2. The MS2 genome encodes four proteins: maturation protein (A-protein), CP, lysis protein and replicase (Figure 1A). Over the years, multiple studies have demonstrated the importance of direct interactions between the 129-residue CP and gRNA in MS2 capsid assembly (13,14,20–26). In particular, single-molecule fluorescence studies have revealed that the hydrodynamic collapse of gRNA during capsid formation in MS2 and other +ssRNA viruses depends on specific interactions between viral gRNAs and their respective CPs (13,14). Using crosslinking and immunoprecipitation (CLIP) coupled with mass spectrometry and RNA footprinting, it was further shown that MS2 assembles via a PS-based mechanism involving direct, induced-fit-type interactions between CP and gRNA (26). In addition to its role in capsid formation, it was shown that CP also induces repression of replicase translation by binding to an RNA stem-loop encompassing the start codon of the replicase gene (21,24,27,28). Importantly, this well-characterized RNA stem-loop serves as the PS involved in initiating capsid assembly (24,25). Finally, in a seminal study, Dai *et al.* have determined a 3.6-Å structure of the mature MS2 virus using cryoelectron microscopy (cryo-EM) (23). Altogether 16 stem loops in the MS2 gRNA (15 contacting a CP dimer and one the maturation protein) were resolved on a single-nucleotide level, implying a stronger interaction with the CP and thus a more central role in capsid assembly (Figure 1B). Three of these stem loops, located in the region between residues 1700 and 1800 in MS2 gRNA, cluster together spatially and bind three neighboring CP dimers. This configuration is, in fact, conducive to nucleating capsid assembly (10,23), with a 19-nt stretch in the middle of these three stem loops being the known PS of MS2 (Figures 1A, B). In further support of an encapsidation mechanism based on multiple PSs, the MS2 binding stem–loops seen in cryo-EM were successfully predicted by the mathematical framework of Hamiltonian paths, relying on such mechanism (29).

As a complement to such mechanistic and structure-based work, several groups have recently developed a powerful new approach for identifying gRNA/CP binding sites in +ssRNA viruses involving RNA-based systematic evolution of ligands by exponential enrichment (SELEX) in combination with bioinformatic analysis (5,15,30–32). Briefly, SELEX is used to generate a pool of RNA aptamer sequences with a high affinity for the CP of choice. A bioinformatic search for matching regions in the viral gRNA is then carried out to identify putative CP binding sites. Shakeel *et al.* have recently performed such an analysis for the picornavirus Human parechovirus 1 (HPeV1) (31). The HPeV1 capsid is composed of 12 pentamers consisting of VP0, VP1 and VP3 subunits, which are cleaved by viral proteases from the single polyprotein (33,34). By performing SELEX against CP pentamers and searching for regions in the HPeV1 Harris strain genome that match the selected aptamer pool, the authors have identified 21 regions in the gRNA that show significant matching with the enriched sequences and are predicted to fold into stem loops with a similar loop motif (31). A similar SELEX-based analysis was recently also performed on Hepatitis B virus (HBV) (30), Hepatitis C virus (HCV) (32), Enterovirus-E (EV-E) (5) and Satellite tobacco necrosis virus (STNV) (35), providing an unprecedented, high-resolution characterization of CP binding sites in different viral species. Finally, CLIP experiments have recently been carried out for several viruses, including MS2 (26), Semliki forest virus (SFV) (36), Sindbid virus (SINV) (37), Venezuelan equine encephalitis virus (VEEV) (38), Chikungunya virus (CHIKV) (39) and Brome mosaic virus (BMV) (40), providing information about possible CP interaction sites in the complete gRNAs. However, the CLIP read-coverage values, typically reported in such studies, are only an indirect proxy of interaction propensity at a given location and must be critically cross-referenced with orthogonal information when interpreted. Overall, the high-resolution MS2 structure, together with SELEX- and CLIP-derived datasets, create now a solid foundation for studying the general principles of packaging in +ssRNA viruses.

Related to this, a recent result concerning nucleic-acid/protein recognition could carry significant implications for understanding specificity in viral packaging. Namely, it was shown that nucleobase-density profiles of mRNA coding sequences (CDSs) in general quantitatively mirror the respective nucleobase-affinity sequence profiles of proteins they encode (41–43). For example, pyrimidine (PYR)-density profiles of CDSs in human match the PYR-mimetic affinity profiles of their autologous proteins with a median Pearson correlation coefficient of *R* = –0.74 (note that matched profiles correspond to a negative Pearson *R* due to the standard way of how affinities are defined) (43). Similar results were also obtained by several computationally derived nucleobase/amino-acid affinity scales, with the opposite behavior observed only in the case of ADE (41,42,44–47). On the basis of such analyses, it was suggested that proteins in general bind to their autologous mRNA CDSs in a complementary, co-aligned manner, especially if unstructured. Importantly, it was suggested that proteins could also bind to non-autologous RNAs in regions where their respective nucleobase-affinity and nucleobase-density profiles match (48). Finally, as these results were based on the analysis of primary sequence profiles, it was suggested that any putative binding would occur in the context of dynamic, liquid-like, multivalent complexes involving unstructured RNA and protein fragments.

Arguably the simplest biological system in which proteins reside in a close proximity of their autologous RNAs are +ssRNA viruses. When applied in this context, the autologous mRNA/protein complementarity hypothesis predicts that in the unstructured state CP will bind directly to the part of the viral gRNA that encodes it, and that this will be reflected in the matching between the CP nucleobase-affinity sequence profiles and the nucleobase-density profiles of its CDS (Figure 1C). More importantly, the hypothesis proposes (48) that CP will also bind to other regions of the viral gRNA in which RNA nucleobase-density and CP nucleobase-affinity profiles match. Here, we directly test these predictions for MS2, leveraging the high-resolution information provided by its cryo-EM structure and assuming that the key contacts are already established in the unstructured state of the biomolecules involved (23). Along with the analysis of gRNA and CP primary-sequence profiles, we develop and apply a linearly additive model to predict site-resolved gRNA/CP interaction energies in the unstructured state from knowledge-based nucleobase/amino-acid affinities and sequence information only (Figure 1D). Finally, we use the model to predict CP/gRNA interaction sites for a comprehensive set of 1082 different +ssRNA viruses and validate its predictions in MS2 and 9 other viruses for which SELEX and CLIP data on the interaction between CPs and complete gRNAs is available (Figure 1E). Our results suggest an important role for interactions between unstructured gRNA and CPs during packaging in +ssRNA viruses and establish a sequence-based computational framework for predicting gRNA/CP interaction sites.

## MATERIALS AND METHODS

### Generation of sequence profiles

The one-dimensional sequence profiles (e.g. nucleobase-density profiles of gRNAs or nucleobase-affinity profiles of CPs) were generated by assigning individual sequence units with numerical values related to the property of interest, e.g. nucleobase-contents of individual gRNA triplets or nucleobase-affinities of individual CP amino acids. The profiles were subsequently smoothed using a window-size of 21 residues/triplets as done before (42,43). Note that when one smooths a given sequence profile using a window size *w* and maps the value in a given window to the central amino-acid or nucleotide in the window, the first and the last $\frac{{( {w - 1} )\ }}{2}$ residues/triplets in the sequence by default cannot be associated with an average value. The nucleobase-affinity profiles were calculated utilizing two different types of nucleobase/amino-acid affinity scales: the experimental PYR-mimetic affinity scale (the polar requirement scale), derived by Woese using substituted pyridines as PYR mimetics (49), and four knowledge-based scales (ADE-affinity, GUA-affinity, CYT-affinity, URA-affinity), derived from high-resolution structures of RNA-protein complexes using a knowledge-based, statistical-potential formalism (42,48).

### Comparison of sequence profiles

Pearson R correlation coefficients were used to compare the smoothed nucleobase-affinity profiles of viral CP sequences of length *l*, against all corresponding smoothed nucleobase-density profiles containing *l* triplets in the respective gRNAs (Figure 1C). Effectively, we slid different CP nucleobase-affinity profiles along the respective gRNA nucleobase-density profiles in steps of 1 nucleotide and evaluated at each point their similarity. It was previously shown that the alignment mapping one amino acid to three nucleotides (one triplet) results in the most significant profile matching, potentially reflecting the optimal way of how unstructured polypeptides and oligonucleotides interact (41–43). In the case of MS2, the CP’s N-terminal methionine and its codon were not included in the analysis as this residue is not present in the mature CP (50). The Pearson *R* for a specific RNA fragment was assigned to its central nucleotide. For CP sequences with an even number of amino acids, the value reported for a given position *i* was the average of the two values that would theoretically correspond to positions *i* – 0.5 and *i* + 0.5. As a consequence of the assignment to the central nucleotide and the preceding smoothing, the first and the last $\frac{{3l - 1}}{2} + 30$ positions for CP profiles of length *l* could not be assigned a Pearson *R*, if *l* is an odd number, or $\frac{{3l}}{2}\ + 30$ positions, if *l* is an even number. For example, the MS2 CP is 129 amino acids long, which means that its nucleobase-affinity profile, smoothed with a window-size of *w* = 21 amino acids, has a length of 109 since the N- and C-terminal 10 amino acids are not associated with a smoothed value (*l* = 129–10-10). Similarly, the first and the last 10 triplets (i.e. 30 nucleotides) in the smoothed MS2 gRNA nucleobase-density profile are not associated with a smoothed value. For these reasons, the first and the last $\frac{{3*109 - 1}}{2} + 30\ = \ 193$ positions in the gRNA are by default not mapped to a Pearson *R*.

### Calculation of relative interaction energy profiles

Similar to the comparison of sequence profiles, the CP sequence is slid along the gRNA sequence in steps of one nucleotide and the theoretical interaction energy determined at each point. For a specific gRNA fragment in question, every amino acid in the protein sequence was aligned to one triplet in the RNA sequence. Subsequently, the sum of the products of nucleobase/amino-acid affinities and the respective nucleobase content of the aligned codon was calculated for all 4 RNA nucleobases (A, G, C, U) (Figure 1D) and the value assigned to the central nucleotide position of that particular fragment, as described above. The resulting profile was then window-averaged using a window-size of 63 nucleotides. As the full CP sequence is aligned to triplets in each sliding window and the resulting profile smoothed afterwards, every nucleotide position in the genome was assigned a value, except for the first and the last $\frac{{3{N_{aa}}}}{2} + 31$ positions for sequences with an even number of amino acids and $\frac{{3{N_{aa}} - 1}}{2} + 31$ for sequences with an odd number of residues *N_aa_*. For example, in the case of MS2 and its 129 amino-acids long CP, all nucleotides in the MS2 gRNA were assigned a relative interaction energy except for the first and the last 224 nucleotides. The theoretical interaction energies were reported either in relative energy units (Figure 3) or as *z*-scores (Figures 3, 5, Supplementary Figure SI5–8) with regard to the distribution of all values obtained at different gRNA positions. Finally, note that the reported energies do not correspond to the absolute binding free energies and should only be used for the relative ranking of different sites.

### Theoretical prediction of gRNA/CP interaction sites

All gRNA positions associated with Pearson Rs or theoretical interaction energies below or equal to a preselected cutoff were treated as the theoretically predicted interaction sites and were compared against their experimentally determined counterparts. In MS2, the latter were derived from either the cryo-EM analysis by Sun and coworkers (23) or CLIP analysis by Stockley and coworkers (26). Specifically, all nucleotides in the 15 high-resolution stem loops that interact with the CP in the MS2 cryo-EM structure (23) were set as the reference binding sites (positions 102–114, 179–200, 593–606, 902–915, 977–990, 1460–1470, 1720–1731, 1747–1763, 1776–1791, 2040–2053, 2374–2387, 2468–2481, 2781–2796, 2840–2852 and 3359–3372). For viruses with gRNA/CP interaction data based on RNA SELEX, the reference binding sites correspond to the gRNA stem loops with either significant or significant and highly conserved matching with the selected anti-CP aptamer sequences and were taken as reported in the original studies in the case of HPeV1 (31) and EV-E (5) viruses. Since the exact locations of those stem–loops were not reported for HBV and HCV, the positions were estimated by taking a 31-nucleotide region centered around loop motifs reported by the authors (30,32). Finally, in the case of STNV, the 1.2-kb-long gRNA encodes just the CP CDS, which covers 47% of the gRNA. Importantly, 18 (Pearson *R*) or 19 (theoretical interaction energies) of the 30 SELEX-detected binding stem loops in STNV are located in the 5′- or 3′-terminal regions for which our predictions cannot be carried out due to technical reasons (see above). For this reason, we have excluded STNV SELEX data from the present analysis.

For CLIP data on MS2, we have explored alternative definitions of reference binding sites by: (i) taking all 54 binding regions of length 41 nucleotides as defined by the authors (26) and (ii) taking the gRNA positions that were among the top 10% according to read coverage. We have assessed the sensitivity of our results to these definitions by systematically exploring alternative lengths of the 54 reported binding regions in the former case or testing a range of different percentage cutoffs in the latter case. For CLIP data on SFV, the top 21 CP binding-sites reported by the authors were set as the reference (36). In the case of all other CLIP results (37–40), the reference binding sites were not explicitly provided by the original authors and were defined analogously to MS2 by taking a given percentage of top gRNA positions according to read coverage, with percentage cutoffs between 1% and 20% explored. Due to the fact that the raw CLIP data was not available for MS2 (26), BMV (40) and CHIKV (39), in those cases the absolute read coverage curves were computationally digitized from the original publications. Finally, the overlap between the MS2 gRNA/CP binding sites seen in the MS2 cryo-EM structure and detected in the CLIP experiments was quantified by using the Jaccard index, defined as the ratio of the size of the intersection of the two sets of genomic positions divided by the size of their union.

### Comparison of predicted interaction sites with experimental data and analysis of statistical significance

To quantify the matching with the experimentally known binding sites, *binding site coverage* (BSC) at a given cutoff was defined as the fraction of nucleotides belonging to the experimentally determined binding sites that were correctly identified in the theoretical prediction. Since not all genomic positions were assigned a predicted value, only those positions which were available in the theoretical profiles were taken into the account. As a consequence, the BSC values range between 0 and 1. The BSC values obtained by analyzing Pearson R or theoretical interaction energy profiles of gRNA and CP sequences were compared against the BSC values obtained for shuffled gRNA or CP sequences, i.e. sequences that were re-ordered at random without changing their composition. Specifically, either the gRNA or the CP sequence was shuffled 1000 times, while the other sequence remained the same. For every shuffled sequence, the Pearson *R* or theoretical interaction energy curve in question was determined in the same way as for the native sequences. The reported *P*-values associated with the BSC at a given cutoff correspond to the fraction of shuffled protein/genome sequences that led to a greater or equal BSC at the same cutoff.

### Prediction of interaction regions for all annotated viral CP sequences

Initially, all+ ssRNA viruses reported in the NCBI Virus Database with complete RefSeq genome sequences and molecule type were considered (1622 viruses; download date: 25 October 2021). For each of the viruses, CP sequences were selected from among their protein and mature peptide sequences using the criterion that the product name contained the terms ‘coat’, ‘capsid’, or ‘core’, while simultaneously not including one of the following terms: ‘precursor’, ‘polyprotein’, ‘readthrough’, ‘read-through’, ‘leader’, ‘duplicate’, ‘homolog’, ‘coat-like’, ‘extension’, ‘extended’, ‘RNA replicase’ or ‘proteins’. For viral species belonging to *Picornaviridae*, the terms ‘VP0’, ‘VP1’, ‘VP3’, ‘VP2’ and ‘VP4’ were additionally included in the search. When no CP sequence could be found in this way, the annotated regions of all viral proteins were also considered, with the same criteria applying to the region name. The set of sequences was further reduced by excluding protein sequences with fewer than 50 amino acids. Moreover, sequences containing ambiguous nucleotides or amino acids were removed from the set. This has led to a total number of 1082 viruses for which CP sequences could be found unambiguously. The theoretical relative interaction energy profiles were calculated for all RNA-protein combinations for each virus and the regions with the lowest 1% or 5% of binding energies reported (Data SI1). Note that whenever these regions were <10 nucleotides apart, they were merged together.

## RESULTS

### MS2 gRNA nucleobase-density profiles match CP nucleobase-affinity profiles at known binding sites

**Figure 2.**
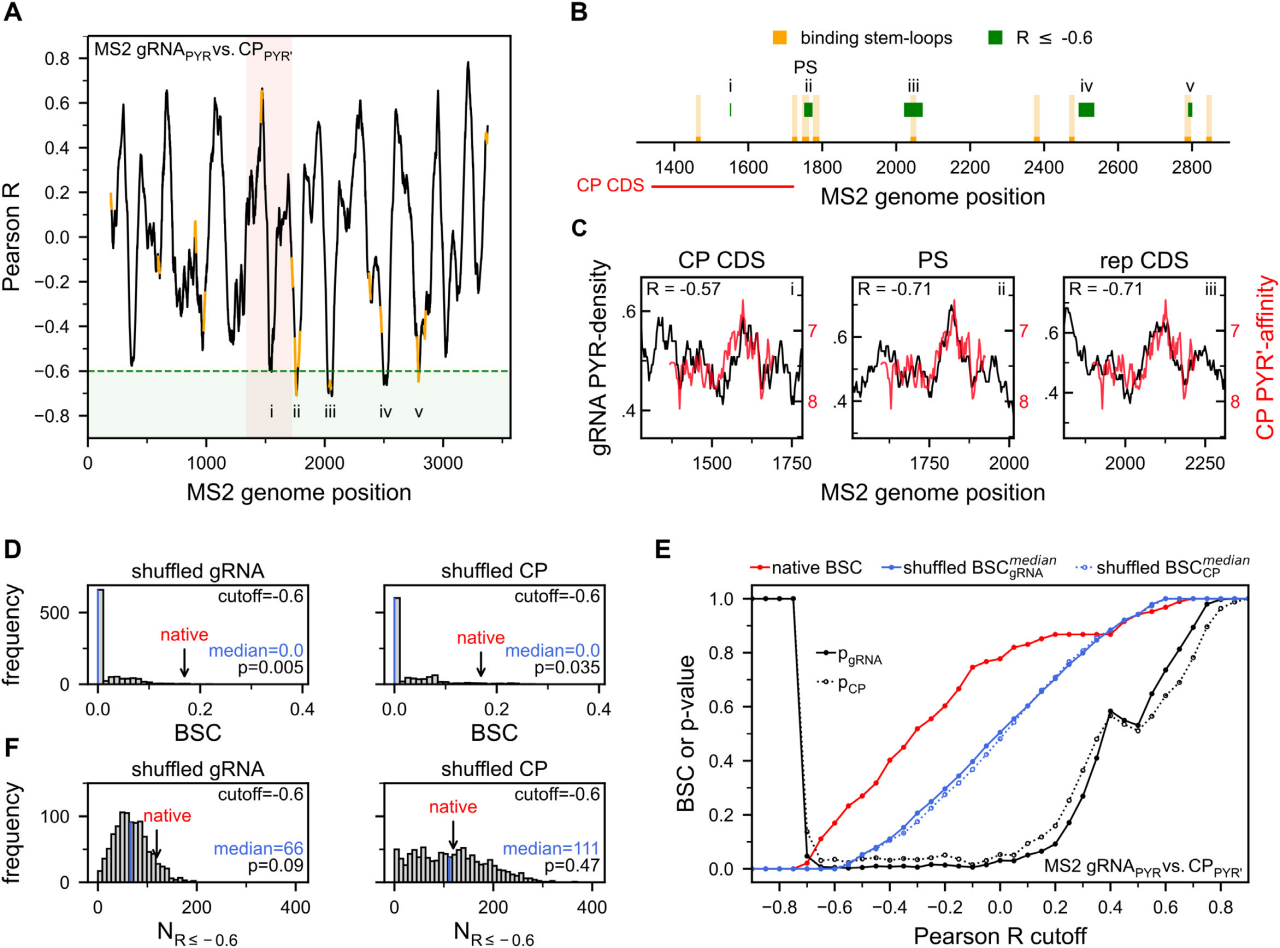
PYR-profile matching captures the location of MS2 gRNA/CP binding sites. (**A**) Pearson Rs between MS2 gRNA PYR-density and CP PYR’-affinity profiles (gRNA_PYR_ versus CP_PYR’_) along the genome (CP CDS in light red). Locations of stem loops that interact with the CP in the MS2 cryo-EM structure are depicted in orange, while individual regions with R ≤ -0.6 (green) are labeled with Roman numerals. (**B**) Locations in MS2 gRNA with *R* ≤ –0.6 overlaid with the locations of cryo-EM CP-binding stem loops (color code and labeling the same as in A). (**C**) Alignment of MS2 gRNA PYR-density and CP PYR’-affinity profiles at different locations in the gRNA including the CP CDS (left), the region surrounding the PS (middle) and the best matching region in the replicase CDS (right). The Pearson Rs resulting from these alignments are shown in the top right corner. (**D**) Distributions of MS2 gRNA_PYR_ vs. CP_PYR’_ BSC at Pearson *R* cutoff of –0.6 for 1000 shuffled MS2 gRNA (left) and CP sequences (right) with medians depicted in blue. BSC obtained for native MS2 gRNA/CP is marked with an arrow. (**E**) Dependence of MS2 gRNA_PYR_ vs. CP_PYR’_ BSC on Pearson *R* cutoff together with the corresponding *P*-values (black). The native BSC is given in red, while the median values of BSC of shuffled gRNA and CP sequences are given in blue. (**F**) Distributions of the number of gRNA positions with *R* ≤ –0.6 for 1000 shuffled MS2 gRNA (left) and CP sequences (right) with medians depicted in blue. The equivalent number obtained for native MS2 gRNA/CP is marked with an arrow. The *P*-values correspond to the fraction of shuffled sequences that led to a greater or equal number of genomic positions with *R* ≤ –0.6.

Woese's polar requirement scale is the only available, experimentally determined scale to capture the interaction propensity of all twenty natural amino acids with nucleobase-like compounds (49). Specifically, the scale, labeled herein as PYR’-affinity, captures the relative interaction propensity of amino acids with the PYR mimetic dimethylpyridine. The values of Pearson R resulting from comparing the MS2 CP sequence PYR’-affinity profile against the PYR-density profile along the MS2 gRNA exhibit a periodic alternation between negative and positive values (Figure 2A). While most R values lie between ±0.5, the most prominent peaks reach up to approximately ±0.75. According to the generalized complementarity hypothesis (48), binding between unstructured RNA and protein is expected to take place in regions with strong negative values of Pearson *R* between the respective nucleobase-density and nucleobase-affinity profiles. In agreement with this prediction, 3 out of the 4 most pronounced peaks with a Pearson *R* ≤ –0.6 coincide directly with the gRNA stem-loops which interact with the CP in the MS2 cryo-EM structure, with the fourth one being located very close to a binding stem–loop (Figures 2A, B). In addition, the fifth strongest peak coincides with the CP CDS where profile matching is *a priori* expected (48). A direct superposition of the MS2 gRNA PYR-density profile with the CP PYR’-affinity profile at these locations reveals a strong, quantitative similarity between the two, with a characteristic peak in PYR-density over a span of ∼150 nucleotides in the gRNA being matched by an equivalent increase in the CP PYR’-affinity over ∼50 amino acids (Figure 2C). Remarkably, the level of matching between the CP PYR’-affinity profile and its CDS PYR-density profile (Figure 2C, left), albeit strong (*R* = –0.57), is superseded by the matching between the CP PYR’-affinity profile and the PYR-density profile surrounding the MS2 PS (Figure 2C, middle, *R* = –0.71) or the PYR-density profiles of several different regions in the replicase CDS (Figure 2C, right, best *R* = –0.71).

How significant is the above overlap of the regions where the MS2 gRNA PYR-density profile matches the CP PYR’-affinity profile with the cryo-EM gRNA/CP binding sites? To address this question, we have compared the degree of overlap between the cryo-EM binding sites and the regions where nucleobase-density and nucleobase-affinity profiles match (i.e. *binding site coverage* or BSC, see Materials and Methods for definition) against two types of randomized background (shuffled gRNA with native CP or shuffled CP with native gRNA), allowing us to estimate the statistical significance of native BSC as a function of Pearson R (Figure 2D). For example, at the cutoff of *R* ≤ –0.6, the BSC is 0.17 for the native gRNA/CP combination with only 5 out of the 1000 shuffled gRNAs in combination with the native CP exhibiting a higher BSC (*P*-value = 0.005, Figure 2D, left). Similarly, at the cutoff of *R* ≤ –0.6 and the native BSC of 0.17, shuffling the CP while keeping the gRNA unchanged also resulted in a significant *P*-value (*P*-value = 0.035, Figure 2D, right). Importantly, the *P*-values in both cases remained stable and low for a wide range of Pearson *R* cutoffs, extending approximately from -0.6 to 0 in both cases, indicating that the above findings are robust and are not limited to a specific cutoff in R (Figure 2E).

Interestingly, while the median number of genomic positions in the predicted interaction sites for the native gRNA was almost twice larger than for the shuffled gRNA and native CP sequences at the *R* cutoff of –0.6 (Figure 2F, left), this number was almost identical in the case of shuffled CP and native gRNA (Figure 2F, right). This suggests that equally strong profile matching somewhere along the gRNA could be obtained for shuffled CP sequences as well, but that the native CP profile simply gives better predictions of the exact locations of the experimentally determined binding sites. Clearly, as the shuffled genomic sequences lead to a smaller number of positions below a given R cutoff, their median BSC is naturally lower than in the case of shuffled CP sequences, explaining the difference in the *P*-values obtained for gRNA versus CP shuffling (Figures 2D and E). If one accounts for this by using the BSC normalized by the number of positions below a given cutoff, similar *P*-values could be obtained for shuffled gRNA and CP sequences (*P*-value = 0.039 for shuffled gRNA versus 0.035 for shuffled CP for *R* ≤ –0.6, respectively). This further supports the claim that the effects seen depend primarily on the location of the positions included in the theoretical prediction and less on their sheer number. Finally, it has to be noted that all of the above analysis was performed on the profiles that were smoothed using a window-size of 21 residues/codons. Although some degree of window-averaging is necessary to obtain strongly negative Pearson *R*s in locations other than the CP CDS, the above *P*-values remain comparable for a wide range of window-sizes (Supplementary Figure SI1).

How does the above analysis translate in the case of other nucleobase-density i.e. nucleobase-affinity profiles? Similar results are obtained when comparing the MS2 gRNA ADE-density profile against the CP ADE-affinity profile (Supplementary Figure SI2). However, in agreement with the previously observed anti-matching of mRNA ADE-density and autologous protein ADE-affinity profiles, our theoretical prediction of binding sites was performed by including all positions that reached a value greater than or equal to a given Pearson *R* cutoff. Indeed, it is the maxima in the Pearson *R* values for ADE comparison which coincide with the cryo-EM binding sites to a noticeable degree with the *P*-values <0.1 for shuffled gRNA and CP sequences for Pearson *R* cutoffs ranging between 0.5 and 0.3 (Supplementary Figure SI2). While no significant binding-site matching could be observed for the remaining knowledge-based nucleobase/amino-acid affinity scales (Supplementary Figure SI2), a common feature of all used affinity scales is that the above analysis leads to a local minimum (maximum for ADE) in Pearson *R* values close to the PS (Supplementary Figure SI2). This becomes apparent if at each gRNA position one adds up the Pearson *R*s obtained for the four standard knowledge-based nucleobase/amino-acid affinity scales (subtracted for ADE), resulting in a clearly defined global minimum at the very location of the MS2 PS (Supplementary Figure SI3).

### Predicted MS2 gRNA/CP interaction energy in the unstructured state points to PS location

**Figure 3.**
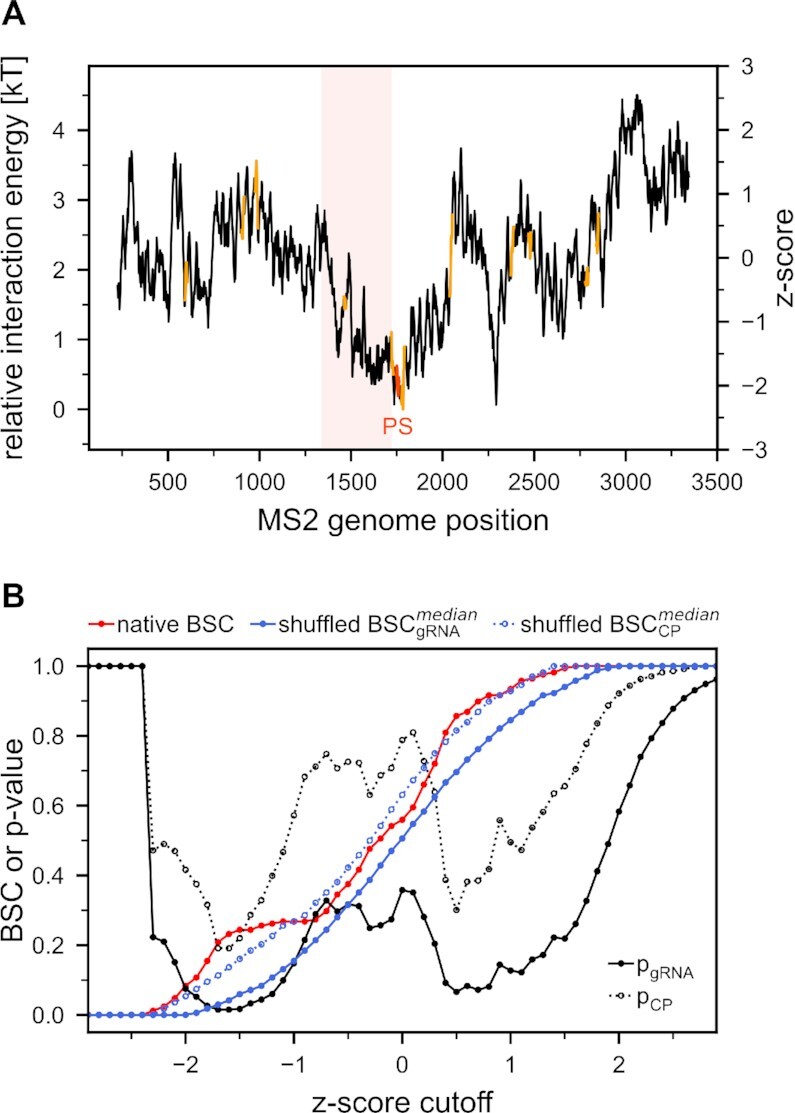
MS2 PS is located in the predicted global minimum of gRNA/CP interaction energy. (**A**) Relative interaction energy of the unstructured CP at every position in the MS2 genome. Positions of stem loops that interact with the CP are marked in orange, while the PS is highlighted in red. The CP CDS is given in light red. (**B**) Dependence of the interaction energy BSC on z-score cutoff together with the corresponding *P*-values (black). The native BSC is given in red, while the median values of BSC for shuffled MS2 gRNA and CP sequences are given in blue.

A comparison of gRNA nucleobase-density profiles and the respective CP nucleobase-affinity profiles by definition involves a single nucleobase type at a time. On the other hand, the binding affinity between unstructured RNA and protein stretches depends on the combined effect of amino-acid affinities for all nucleobase types at a given location. For this reason, we have developed a linearly additive model in which the relative interaction energy between the CP and a given gRNA fragment is equal to the sum of all pairwise nucleobase/amino-acid affinities obtained using a knowledge-based formalism (42), after the two polymers are aligned (Figure 1C, see Methods for details). Following our previous work, the alignment is performed such that one amino acid always interacts with three consecutive nucleobases and the resulting energy profile is smoothed using a 63-nucleotide averaging window (42,43,48). Importantly, the thus derived profile of predicted relative interaction energies of the MS2 CP along its gRNA exhibits a funnel-like shape, attaining a deep minimum, i.e. the highest binding propensity, precisely at the position of its PS, and also including the CP CDS among strong minima (Figure 3A). Comparing the BSC obtained using the relative interaction energy predictions against shuffled controls at various *z*-score cutoffs reveals that for a range of *z*-scores between –1.8 and –1.3 the obtained *P*-values for shuffled gRNA sequences reach <0.05. Interestingly, no strong statistical significance is observed for shuffled CP sequences (Figure 3B). This suggests that proteins of a similar amino-acid composition and length as CP could bind equally well to the MS2 PS, irrespective of their exact sequence, with the wild-type gRNA sequence alone being largely responsible for defining the binding sites. Finally, while some sequence averaging is required to obtain well-defined minima in the interaction energy profile, the BSC and the resulting *P*-values are comparable for a large set of window sizes (Supplementary Figure SI4), similar to the analysis of Pearson *R* values.

### Theoretical prediction of gRNA/CP interaction sites in 1082 +ssRNA viruses

**Figure 4.**
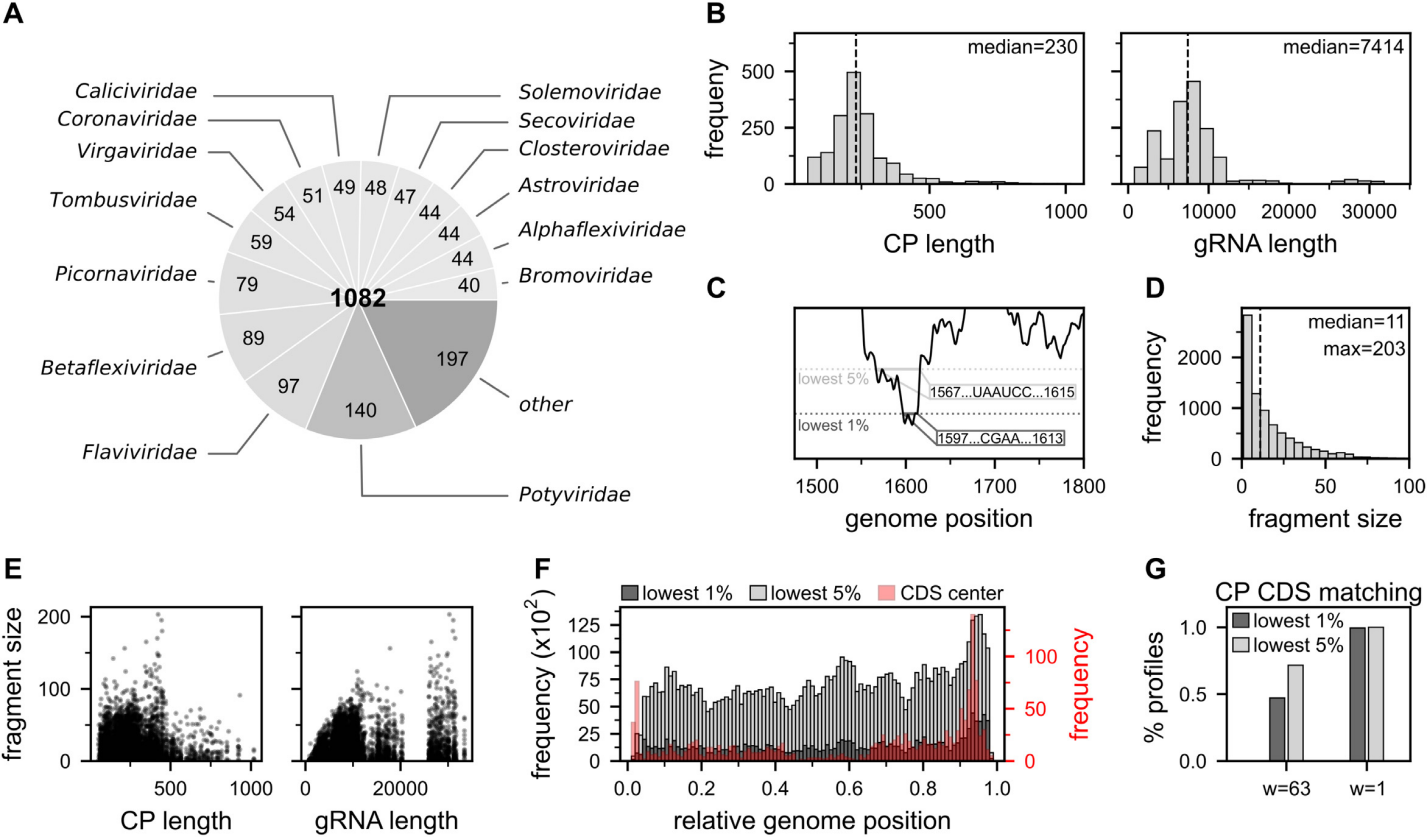
Prediction of gRNA/CP interaction sites for +ssRNA viruses. (**A**) Number of viruses for which theoretical predictions were reported, grouped by viral families. (**B**) Distribution of CP and gRNA sequence length for the set of sequences used in the analysis. (**C**) For every calculated gRNA/CP interaction energy profile, the regions in the viral gRNA which correspond to the lowest 1% or the lowest 5% of predicted interaction energies are reported in Dataset SI1. (**D**) Distribution of sizes of the predicted fragments associated with the lowest 1% of interaction energies. (**E**) Fragment size for the lowest 1% predicted interaction energies depending on CP and gRNA sequence length. (**F**) Relative gRNA positions of the lowest 1% (dark gray) or 5% (light gray) of predicted interaction energies for the entire set of analyzed +ssRNA CP/gRNA sequences. Locations of the CP CDS center are shown in red. (**G**) Percentage of analyzed theoretical interaction energies profiles, which have at least one overlapping position between the regions of the lowest 1% (dark gray) or the lowest 5% (light gray) predicted interaction energies and the CP CDS, for window-sizes *w* = 63 or *w* = 1.

As our linear model for relative energy estimation was able to successfully capture the high-affinity MS2 gRNA/CP binding site where capsid assembly is initiated, i.e. its PS, we have employed it to predict gRNA/CP interaction sites for a comprehensive set containing 1082 +ssRNA viral species from 40 different families (Figure 4A). The lengths of the analyzed gRNA sequences spanned a range between ∼800 to 33 000 nucleotides, with a median length of 7414 (Figure 4B), while the CP sequence lengths ranged between 50 (the lower bound set by us) and ∼1000 residues, with the median length of 230. For every possible gRNA/CP combination in a given viral species (relevant for multi-segmented viruses and viruses with multiple CP sequences), we report the genomic fragments which correspond to either the lowest 1% or the lowest 5% theoretical interaction energies over the entire gRNA (Figure 4C, Supplementary Data SI1). The median length of the fragments predicted by the lowest 1% theoretical interaction energies is 11 nucleotides, with the largest ones reaching up to ∼200 nucleotides (Figure 4D). While the maximum fragment sizes predicted in this way are naturally limited by the gRNA length, a wide range of predicted fragment sizes is still covered for large gRNA sequences (Figure 4E). Interestingly, while the predicted regions are found in different locations in the entire gRNA, a clear preference for the 3′ termini is observed. This finding is very much related to the fact that the CP CDS regions tend to be located at the 3′ ends of the gRNAs for the viruses analyzed here (Figure 4F). As expected, the majority of the analyzed sequences show an overlap between the predicted fragments and the CP CDS. In particular, the predicted fragments overlap with the CP CDS for approximately 50% of the calculated profiles in the case of regions corresponding to the lowest 1% of predicted interaction energies and ∼70% in case of the regions corresponding to the lowest 5% of predicted energies. However, as already discussed for MS2, while averaging is necessary to obtain strong minima in the gRNA (other than at the CP CDS), the CP CDS tends to overlap with a strong minimum independent of averaging. This is in agreement with the fact that when the theoretical interaction energy profiles are not averaged, these numbers shift to 100% for regions corresponding to the lowest 5% of interaction energies and to 99.5% for regions corresponding to the lowest 1% (Figure 4G) of interaction energies.

### Predicted gRNA/CP interaction energy in the unstructured state captures SELEX interaction sites

**Figure 5.**
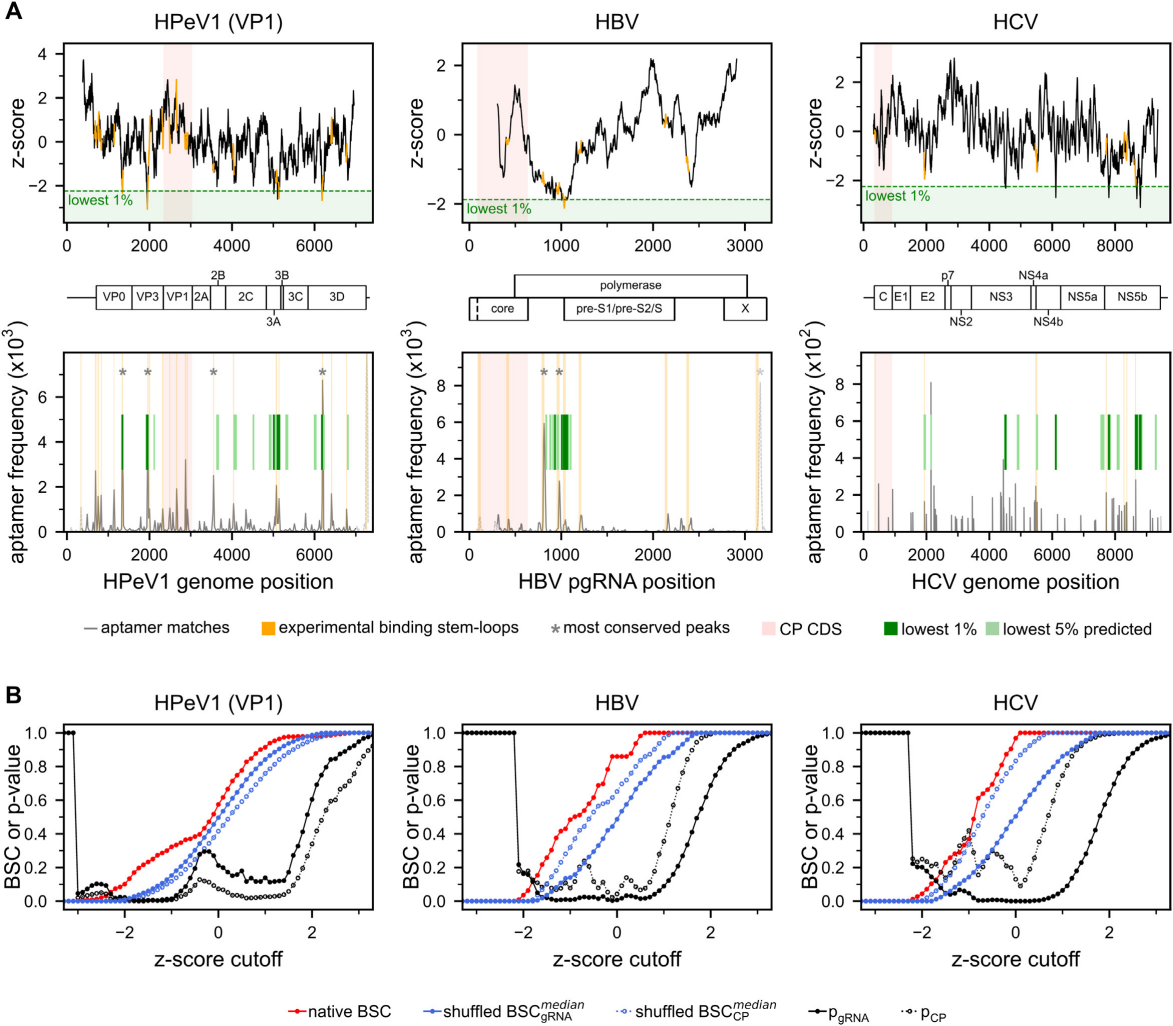
Minima in predicted interaction energy match the gRNA/CP interaction sites as determined by SELEX. (**A**) *top row* Z-scores of the predicted relative interaction energy for the unstructured CP along the gRNAs for HPeV1, HBV and HCV. For HPeV1, the interaction energies of VP1 are shown, while the interaction energies of VP0 and VP3 can be found in Supplementary Figure SI5. Positions of the stem loops identified via SELEX as interacting with CP are given in orange. *bottom row* Regions corresponding to the lowest 1% and lowest 5% predicted interaction energies are depicted as dark and light green bars, respectively. Experimentally determined anti-CP aptamer matches are shown in gray, while the locations of stem loops identified via SELEX as interacting with CP are given in orange (5,30,31). Peaks in the aptamer matches that are most conserved between strains are marked with stars. Dashed lines indicate regions that are outside of the predictable range in our model. (**B**) Dependence of the interaction-energy BSC on *z*-score cutoff together with the corresponding *P*-values (black) for HPeV1 (VP1), HBV and HCV. The native BSC is given in red, while the median values of BSC for shuffled gRNA and CP sequences are given in blue, analogous to Figure 3. Positions that were set as the reference for calculation of BSC correspond to orange regions in A).

In order to provide validation for the above predictions, we have analyzed the gRNA/CP interaction sites as determined by SELEX for Human parechovirus 1 (HPeV1), Hepatitis B virus (HBV), Hepatitis C virus (HCV) and Enterovirus-E (EV-E), four systems for which such high-resolution, site-resolved data is available (5,30–32). Remarkably, the regions with the lowest 1% theoretical interaction energies of VP1 along the HPeV1 Harris genome directly coincide with the location of three out of four most conserved binding stem-loops detected experimentally as being key CP binding regions (Figure 5A, left). Further experimental binding stem-loops are captured if one considers the regions with the lowest 5% of theoretical binding energies (Figure 5A, left). Importantly, the global minimum in our predicted interaction energy profiles is located in the VP3 CDS, where indeed the highest level of conservation was observed (Figure 5A, left) (31). This global minimum is also identified if one considers the theoretical interaction energies of VP3 along the gRNA (Supplementary Figure SI5). Taking the positions of the 21 stem loops as a reference for the calculation of BSC and comparison to shuffled sequences leads to significant *P*-values (*P* < 0.01) for a wide range of *z*-score cutoffs for both VP1 (Figure 5B, left; Table 1) and VP3 (Supplementary Figure SI5; Table 1). In contrast, no extensive overlap between the experimentally determined stem loops and the minima in the theoretical interaction energies of VP0 along the HPeV1 Harris strain genome is obtained (Supplementary Figure SI5), in agreement with the fact that gRNA capsid contacts are established with VP1 and VP3, while VP0 mainly is involved in the interaction between capsid pentamers (31,33).

**Table 1.** Comparison between predicted gRNA/CP interaction sites and those observed by cryo-EM or SELEX. Analyzed viruses and their CPs, together with the method used to study the gRNA/CP interaction. In the column labeled ‘IS in CDS’ (interaction site in coding sequence), ‘yes’ corresponds to the fact that there is at least one experimentally determined interaction site in the CP CDS, as defined in the Methods section. Stars indicate the level of statistical significance for BSC analysis for shuffled gRNA and shuffled CP sequences according to the legend given below the table

				Profile matching		Interaction energy	
Species	Method	Protein	IS in CDS	shuffled_gRNA_	shuffled_CP_	shuffled_gRNA_	shuffled_CP_
MS2	Cryo-EM	CP	Yes	PYR’***, -ADE***	PYR’**, -ADE*	**	-
HPeV1	SELEX	VP3	Yes	PYR’*, URA***	PYR’*, URA***	***	***
		VP1	Yes	PYR’*, URA***	PYR’**, URA**	***	***
HBV	SELEX	CP	Yes	URA***, CYT**, GUA*	URA***, CYT**, GUA**	***	*
HCV	SELEX	core	Yes	GUA***	GUA***	**	-
EV-E	SELEX	VP0	Yes	PYR’***	PYR’***	-	-
		VP3	Yes	-	-	*	**
		VP1	Yes	-	-	***	***

*At least one *R* cutoff $ \le$ -0.5 (*R* cutoff $ \ge$ 0.5 for -ADE) / *z*-score cutoff $ \le$ –1 with *P*-value < 0.1.

**At least one *R* cutoff $ \le$ -0.5 (*R* cutoff $ \ge$ 0.5 for -ADE) / *z*-score cutoff $ \le$ –1 with *P*-value < 0.05.

***At least one *R* cutoff $ \le$ -0.5 (*R* cutoff $ \ge$ 0.5 for -ADE) / *z*-score cutoff $ \le$ –1 with *P*-value < 0.01.

Similarly, the theoretical interaction energies of the HBV CP and pre-genomic RNA (pgRNA) display a wide minimum, which significantly overlaps with the locations of binding stem-loops identified by SELEX (Figure 5A, middle; Table 1). Although HBV is a para-retrovirus (DNA virus), Stockley and coworkers have argued that the RNA SELEX-based approach is applicable for the identification of putative PSs in this case as well, given that these viruses initially package pgRNA, a positive-sense RNA form of the genome. Indeed, they were able to identify multiple regions in the pgRNA which significantly match the selected aptamer sequences and simultaneously exhibit a high level of conservation between different HBV strains. By analyzing peak regions that were at least 80% conserved between the tested strains, they identified nine predicted stem-loops in the pgRNA, with an RGAG motif in the loop region (30). Importantly, the wide minimum in the theoretical interaction energy profile overlaps with two peak regions that were among the top three in terms of conservation and aptamer matching frequency (Figure 5B, middle), while the third peak with the highest aptamer frequency is located at the very 3′ terminus where our algorithm by design cannot make predictions. In addition to that, two well-defined minima in theoretical binding energy reside close to the reported stem loops. This matching is significant compared to shuffled pgRNA (*P* < 0.01) and CP (*P* < 0.1) sequences (Figure 5B, middle; Table 1) for a range of *z*-score cutoffs.

A significant overlap with the stem loops identified via SELEX is also observed for the relative interaction energies of the HCV core protein along the respective gRNA (32) (Figure 5A, right; Table 1). In particular, the general regions with a low relative interaction energy cluster around the locations where indeed a high density of experimental peaks was detected (Figure 5A, right), yielding significant *P*-values (*P* < 0.05) for shuffled gRNAs and a range of *z*-score cutoffs. Finally, the theoretical predictions obtained by relative interaction energies for the CP subunits of EV-E (5) also show statistically significant matches with the experimental SELEX data for VP1 (*P* < 0.01 for both shuffled gRNA and CP) and VP3 (*P* < 0.1 for shuffled gRNA and *P* < 0.05 for shuffled CP), although these are limited to the most stringent z-score cutoffs only (Supplementary Figure SI6, Table 1). On the other hand, there are no significantly matched binding sites for EV-E VP0. However, in contrast to parechoviruses, EV-E VP0 is further cleaved into VP2 and VP4 subunits late in assembly, as seen for most other *Picornaviridae*. While in HPeV1 the gRNA/CP contacts are established with residues of VP1 and VP3, the gRNA/CP interactions in EV-E include residues of VP2 and VP4 as well (5,31,34).

Sequence profile comparison also reveals significant overlap between the SELEX-derived binding stem loops and locations where select gRNA nucleobase-density profiles match CP nucleobase-affinity profiles for most of the analyzed viruses (Table 1). For example, for HPeV1 a particularly good agreement is seen between the experimentally determined stem loops and regions where VP1 and VP3 URA-density matches gRNA URA-density. At the Pearson R cutoff -0.5, the BSC is approximately 0.21 for VP3, with no shuffled CP or gRNA sequence having the same or higher BSC (*P*-value < 0.001, Table 1). Similarly, HBV CP URA-affinity matches pgRNA URA-density preferentially at locations containing SELEX identified binding stem loops (BSC = 0.33 at *R* cutoff –0.5, with *p*_gRNA_ = 0.006 and *p*_CP_ = 0.008), with additional matching observed for CYT and GUA nucleobase-density and nucleobase-affinity profiles (Table 1). Finally, HCV core GUA-affinity and gRNA GUA-density shows a significant matching to the experimentally determined stem loops (Table 1).

### Comparing theoretical predictions to CLIP gRNA/CP interaction data

Analysis of BSC and the associated *P*-values has also revealed significant matching (*P*-value < 0.1 and *z*-score $ \le$ –1) between our predictions based on interaction energy and the experimental CLIP data for VEEV, SINV, CHIKV and BMV (Supplementary Figure SI7, SI8, Supplementary Table S1). However, in all of these cases, matching was observed for specific cutoffs only and, as a whole, was associated with weaker statistical significance as compared to the results obtained for the MS2 cryo-EM structure or the binding sites derived by SELEX. We have additionally explored different cutoffs as the definition of CLIP reference binding sites ranging between the top 1% and 20% in read coverage, but without a significant change in results (Supplementary Figure SI9). Finally, a more detailed analysis of SFV, for which the binding sites were more clearly defined by Brown *et al.* (36), also did not reveal any statistically significant trends. The principal challenge is that the reported heights of CLIP read coverage peaks do not directly correspond to the strength of binding at a given site. As a case in point, a detailed comparison of the cryo-EM structure of the MS2 virus with the respective MS2 CLIP data revealed only a limited agreement between the two. For example, the CLIP-defined CP-binding sites in MS2 overlap with the cryo-EM binding stem loops with a Jaccard index of only 7.5% (see Materials and Methods for definition; Supplementary Figure SI10). Moreover, even this limited agreement between the two is largely due to the fact that the CLIP-defined binding sites cover a large fraction (56.4%) of all nucleotides in the MS2 genome (Supplementary Figure SI10). Namely, if one takes just the two flanking nucleotides on either side of the CLIP peaks to define the binding sites, thus approximately equalizing the number of binding nucleotides in CLIP and cryo-EM experiments, the Jaccard index drops further to only 4.3%. Finally, if one uses the BSC analysis and asks how well the CLIP-defined binding sites agree with the 15 binding stem loops seen in the MS2 cryo-EM structure, one obtains statistically unsignificant *P*-values (>0.1) regardless of how many nucleotides flanking the CLIP crosslink sites are taken to define the binding stem loops. This suggests that the raw sequencing reads from CLIP experiments provide a less precise picture of the exact gRNA/CP binding sites as compared, for example, to SELEX data. Despite the general qualitative agreement with our predictions, the CLIP results may therefore be less well suited for the quantitative comparison required.

## DISCUSSION

**Figure 6.**
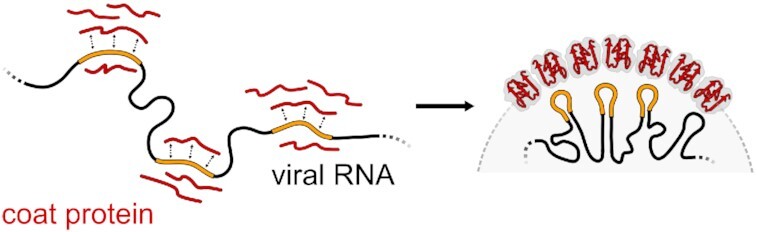
Binding in the unstructured state and viral capsid assembly. In the unstructured state, dynamic interactions between gRNA and CP in select regions of high mutual binding affinity could contribute to a local accumulation of CPs. These interactions could then guide the co-folding of gRNA and CP and provide a foundation for subsequent encapsidation.

We have used multiple +ssRNA viruses to show that different gRNA nucleobase-density profiles tend to mirror the respective CP nucleobase-affinity profiles at the experimentally known gRNA/CP binding sites. Moreover, we could show that many of these sites, including known PSs, reside in deep global minima of the gRNA/CP interaction energy as evaluated using a linearly additive model and an assumption of extensive structural disorder. Overall, these results point to an important contribution of gRNA/CP interactions in the unstructured state to capsid assembly in +ssRNA viruses. Namely, multivalent, dynamic interactions in the unstructured state, which are arguably well captured by our primary-sequence analysis, could increase the local concentration of CPs at proper sites along gRNA in the early stages of capsid assembly. This, in turn, could also modulate the folding landscape of both CPs and the gRNA and guide the process of capsid assembly (Figure 6). It is unlikely that co-aligned binding with the gRNA over the complete CP sequence is ever realized, but we do expect that the strong compositional and energetic biases revealed by the analysis of complete sequences could lead to localized interactions between multiple shorter stretches. Importantly, our results highlight the relevance of more diffusive, long-range compositional patterns in both gRNA and CP sequences in establishing interaction specificity, complementing previous analyses, which focused on more sharply defined motifs (51). Finally, experimental evidence analyzed herein points to widespread interactions between CPs and their coding regions in the gRNA (Table 1; Supplementary Table SI1). Similarly, our predicted interaction energies frequently include the CP CDS as one of the top putative CP-binding sites (Figure 4G). These findings are consistent with a recent proposal that proteins in general interact with both their autologous mRNA coding sequences and other compositionally similar, non-autologous RNA regions, reflecting the driving forces behind the origin of the universal genetic code. Future research should shed further light on the exciting possibility that the specificity in gRNA/CP recognition in +ssRNA viruses may in part be related to the structure of the genetic code.

Recently, Wimmer and coworkers have used computer design in combination with chemical synthesis to design variants of poliovirus type 1 containing thousands of synonymous mutations in the ORF of the virus (52). They could show that many of the designed variants underwent successful packaging and remained viable despite such massive recoding and, importantly, despite a major change in the nature and the position of stem loops in the gRNA. The authors have interpreted this as evidence against the idea that multiple, sequence-degenerate PSs may be essential for packaging in +ssRNA viruses. Our results, however, provide a new perspective on this interpretation. Namely, the structure of the universal genetic code ensures that recoding of RNA sequences using synonymous mutations typically results in largely unchanged sequence nucleobase-density profiles (53). This, in turn, implies that no degree of gRNA recoding will markedly affect its degree of matching with the CP nucleobase-affinity profiles, i.e. the nature or the location of the CP interaction hot spots along the gRNA. In other words, the essence of PSs may reside not necessarily in their secondary structure features, but rather in their nucleobase-density patterns, which tend to be preserved upon synonymous recoding.

Our prediction of gRNA/CP interaction sites relies exclusively on the compositional features of primary sequences of gRNA and CP and does not explicitly account for any contributions at the secondary, tertiary or quaternary structure level. Conceptually, it is very much related to the way the interaction sites are detected in SELEX, especially on the gRNA side, i.e. via an enrichment of short, linear RNA fragments, which interact with the CP in question, and a search for them in gRNA. This fundamental similarity could explain in part the significant matching between our predictions and SELEX data. However, it is also possible that the features captured at the primary-sequence level also dominate the interactions at higher levels of structural organization, even in the folded state. The primary sequence in both proteins and RNA determines their secondary, tertiary and quaternary structure. It is, therefore, possible that the biases seen at the primary sequence level may remain relevant even in the context of folded partners, as frequently also assumed when interpreting SELEX experiments. In fact, cryo-EM/X-ray analysis of some of the viruses studied here has revealed that the CPs are capable of binding the structural motifs detected by SELEX even in the context of folded gRNAs (5,31). What is more, +ssRNA CPs often exhibit extensive intrinsically disordered regions (54,55) and/or extended structural motifs such as β-strands, while gRNA interacts with CPs mostly via single-stranded loops. In both of these contexts, linear sequence information may be critical for determining interaction specificity. Finally, it is possible that the binding in the unstructured state may be relevant at select sites only, such as the PSs during early interactions, while other sites may rely on alternative mechanisms.

When comparing different CP profiles with the respective gRNA profiles, we have assigned the Pearson R or the interaction energy in question to the very center of the local gRNA fragment used. This choice, based on symmetry and ease of implementation, could have a major impact on how well the real interaction sites are predicted. It is, in fact, possible that the final binding sites are shifted away from where the initial contacts between unstructured partners are established. For example, in the case of the only conserved peak in HPeV1 virus that was not correctly predicted by our binding energy analysis (Figure 5A, left), our prediction was shifted by approximately 100 nucleotides towards the 3′ end of the gRNA. We surmise that this could be an example of the situation where just one fragment of the whole CP sequence or the gRNA sub-sequence in question is relevant for binding. In addition to the difficulties associated with defining the location of the CP/gRNA interaction sites, other open challenges include a proper treatment of secondary, tertiary or quaternary structure considerations on the side of both gRNA and CP in our predictions, the issue of appropriate energy cutoffs used to define the interaction sites and the uncertainties associated with the linear addition of nucleobase/amino-acid relative binding free energies in determining the relative interaction affinity. Future work should provide a more detailed perspective on these challenges.

Although the comparison between our predictions and CLIP data has revealed significant agreement, it should be strongly emphasized that CLIP read coverage is only an indirect proxy of the frequency/strength of interaction at a given location and multiple factors could make a difference. For example, different gRNA fragments could crosslink with CP to different extents regardless of their mutual affinity, or could be differently affected by the non-specific background interactions. As a case point, the well-defined PS in VEEV could not at all be captured by an increased read coverage in CLIP (38), while the known PS in SFV was not in the top 20 binding sites detected by read coverage in CLIP (36). Finally, in cases where no explicit binding sites were identified by the authors of the original studies, we were limited to a simplistic definition of gRNA/CP interactions sites as a fixed percentage of the top locations in the absolute read coverage reported (e.g. top 10% as shown in Supplementary Figures SI7, SI8, Supplementary Table SI1 but other values did not make a significant difference, see Supplementary Figure SI9**)**. For all of these reasons, we believe that the high-resolution cryo-EM data in the case of MS2 and SELEX-based information on binding in the case of HPeV1, HBV, HCV and EV-E provide a more quantitative foundation for comparing with our predictions and have, therefore, put more emphasis on it here.

In summary, we hope that our results will provide a new perspective on viral capsid assembly and will inspire future work along both experimental and theoretical directions to shed more light on the foundation of this key biological phenomenon.

## DATA AVAILABILITY

Data related to gRNA/CP binding-site predictions in +ssRNA viruses is provided in Data SI1.

## Supplementary Material

gkac202_Supplemental_Files
