# Supplementary material for: Compositional complementarity between genomic RNA and coat proteins in positive-sense single-stranded RNA viruses
Source: Nucleic Acids Res. 2022 Mar 31;50(7):4054–67. doi: 10.1093/nar/gkac202 (PMC9023274; doi:10.1093/nar/gkac202)
Supplement: gkac202_Supplemental_Files [file gkac202_supplemental_files.zip › SI_NAR_01701_Z_2021.pdf]

Supplementary Information

Compositional complementarity between genomic RNA and coat proteins in positive-sense single-stranded RNA viruses

Marlene Adlhart[1], Florian Poetsch[2], Mario Hlevnjak[3], Megan Hoogmoed[1], Anton A. Polyansky[1] &

Bojan Zagrovic[1*]

[1]Department of Structural and Computational Biology, Max Perutz Labs, University of Vienna, Campus Vienna Biocenter 5, A-1030, Vienna, Austria

[2]Institute for Physiology and Pathophysiology, Center for Medical Research, Johannes Kepler University of Linz, Huemerstraße 3-5, 4020 Linz, Austria

[3]Division of Molecular Genetics, German Cancer Research Center (DKFZ), Im Neuenheimer Feld 580, 69120 Heidelberg, Germany

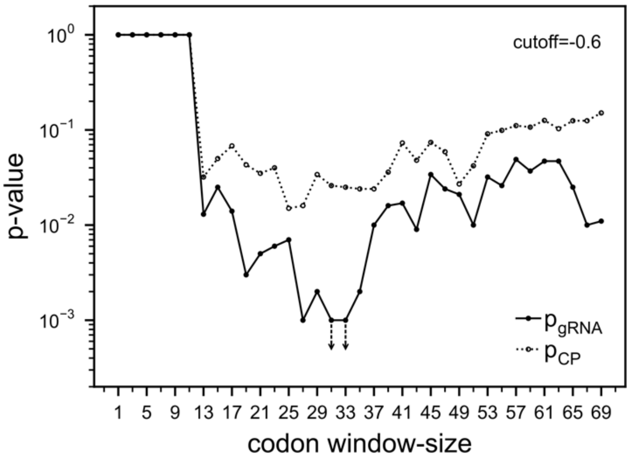

Figure SI1. The p-values for MS2 gRNA$_{PYR}$ vs. CP$_{PYR'}$ BSC at Pearson R cutoff -0.6 for different smoothing window-sizes. The p-values for BSC at Pearson R cutoff -0.6 are depicted for shuffled MS2 gRNA (solid line) and shuffled MS2 CP sequences (dashed line). Arrows indicate p-values < 10^{-3}.

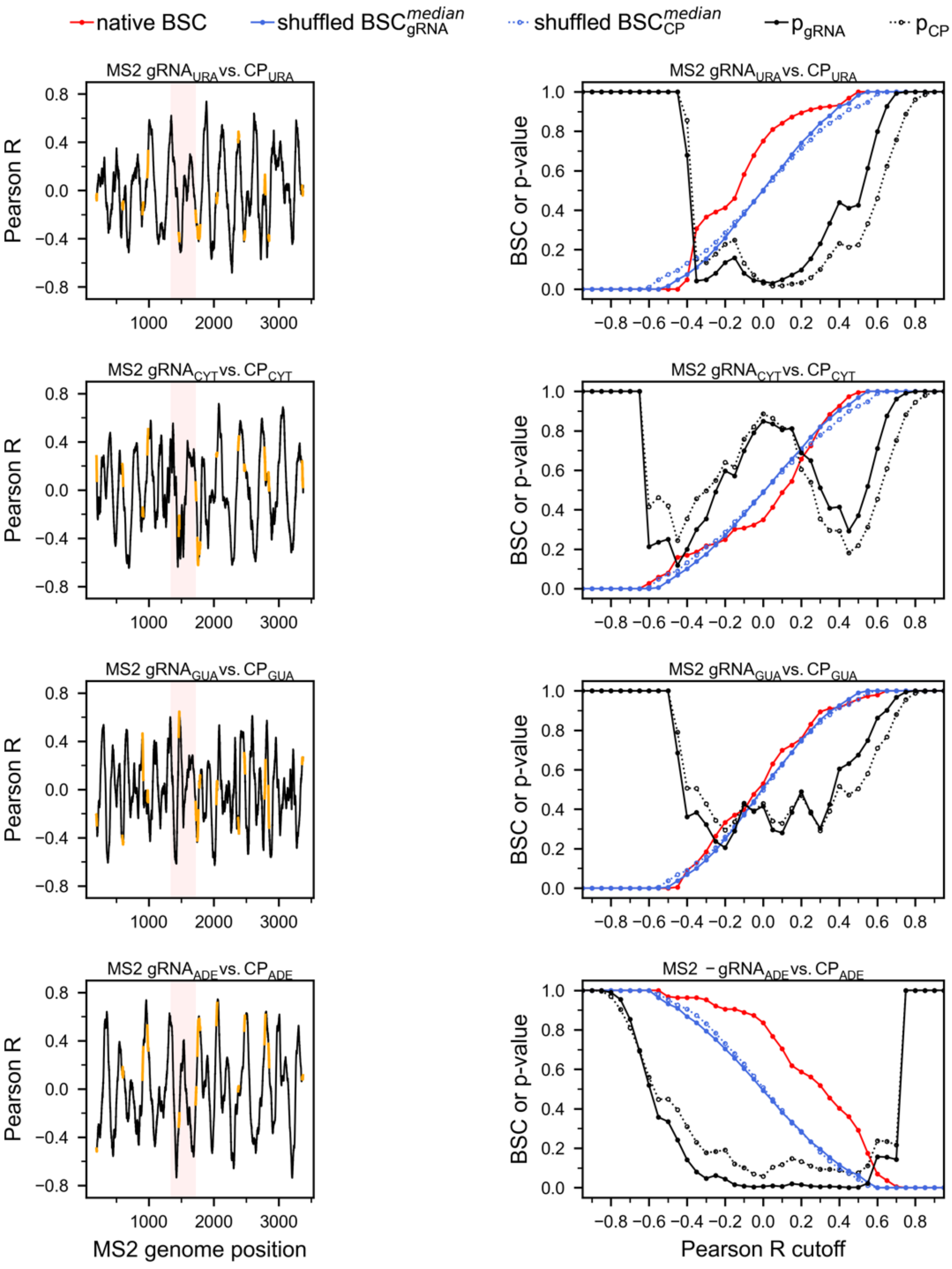

Figure SI2. Profile matching for different MS2 gRNA nucleobase-density and CP nucleobase-affinity profiles. *(left)* Pearson Rs between MS2 gRNA nucleobase-density and the corresponding CP nucleobase-affinity profiles for different nucleobase types. The locations of stem loops that interact with the CP in the MS2 cryo-EM structure are depicted in orange. The CP CDS is highlighted in light red; *(right)* dependence of BSC on Pearson R cutoff together with the corresponding p-values (black) for different profiles. The native BSC is given in red, while the median values of BSC of shuffled gRNA and CP sequences are given in blue. Note that for -gRNA$_{ADE}$ vs. CP$_{ADE}$, all positions that reached a value greater or equal than the given Pearson R were considered as theoretical prediction of interaction sites.

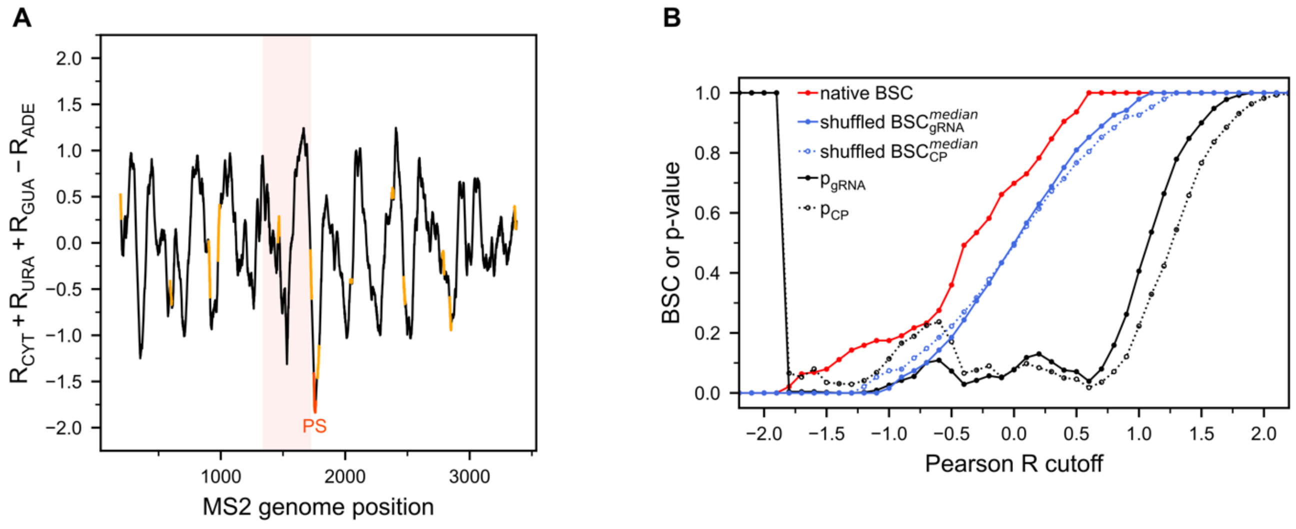

Figure SI3. Combining Pearson R values obtained for all four nucleobase types results in a global minimum at the MS2 PS. A) At each genomic position, the Pearson Rs between MS2 CP nucleobase-affinity and MS2 gRNA nucleobase-density for all four knowledge-based nucleobase/amino-acid affinity scales are combined by adding up the values obtained for GUA, URA and CYT and subtracting the value obtained for ADE (black). CP-binding stem-loops are depicted in orange and the MS2 PS is highlighted in red. The CP CDS is highlighted in light red. **B)** Dependence of BSC on Pearson R cutoff for the combined Pearson R profile, together with corresponding p-values (black). The native BSC is given in red, while the median values of BSC of shuffled MS2 gRNA and CP sequences are given in blue.

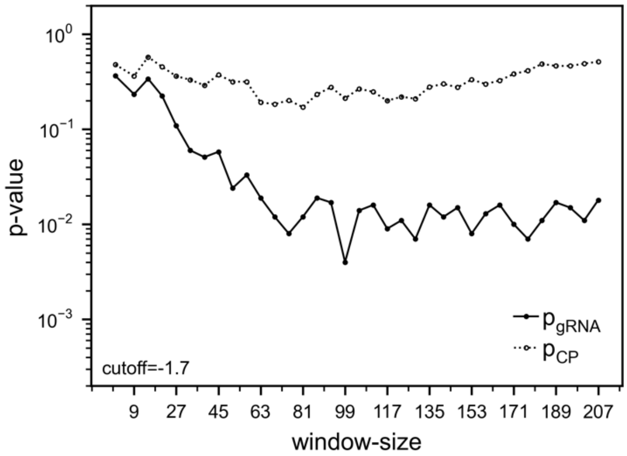

Figure SI4. The p-values for the interaction-energy BSC for MS2 CP along the gRNA at z-score cutoff -1.7 for different nucleotide smoothing window-sizes. The p-values for BSC at z-score cutoff -1.7 (approximately corresponding to the top 5% in the native profile) are depicted for shuffled gRNA (solid line) and shuffled CP sequences (dashed line).

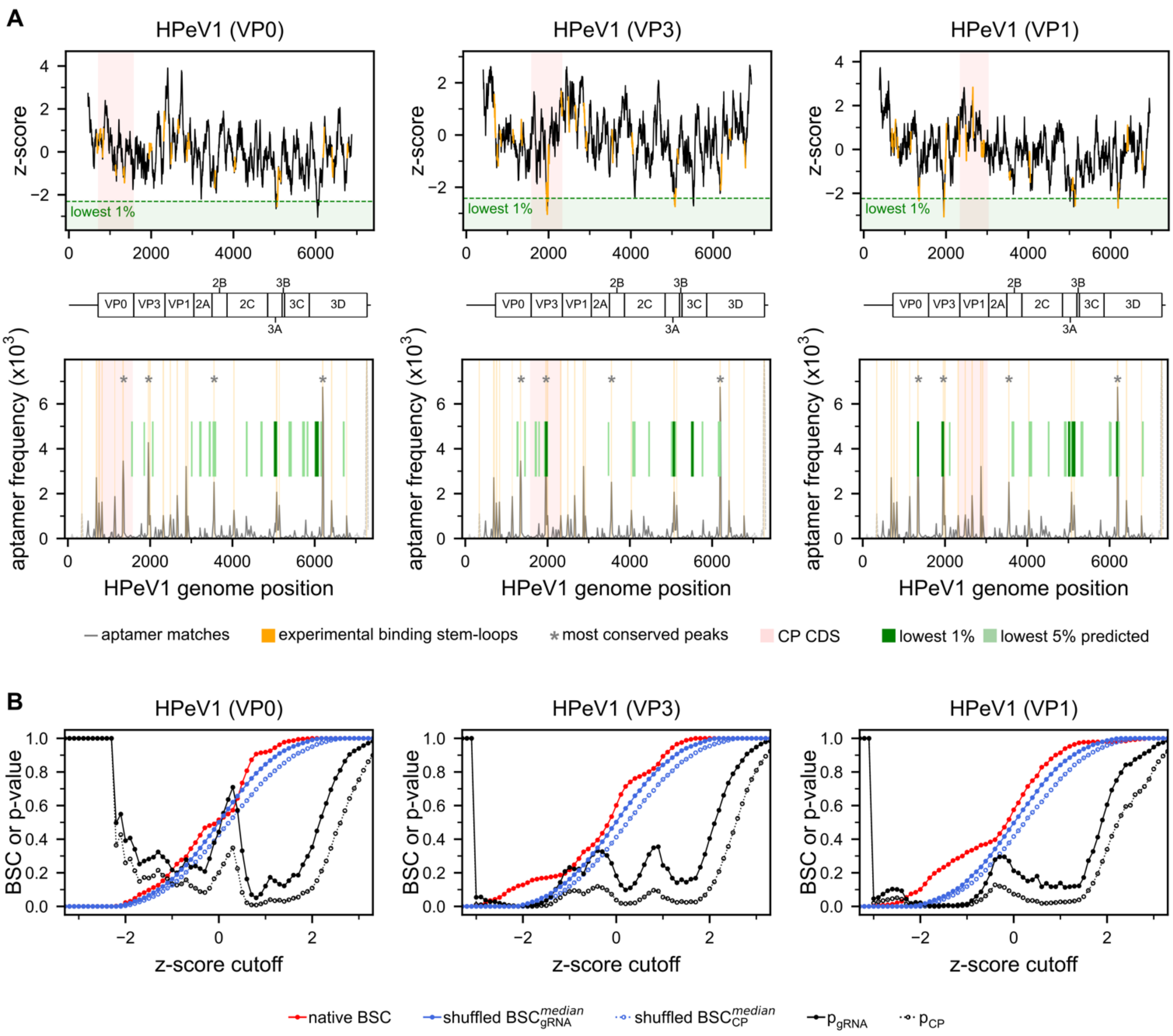

Figure SI5. Theoretical interaction energies of the HPeV1 capsid subunits along the genome and statistical analysis of BSC. A) *(top row)* z-scores of the predicted relative interaction energy for VP0, VP3 and VP1 along the HPeV1 Harris strain genome. The CDS of the corresponding CP subunit is highlighted in light red. Positions of the stem loops identified via SELEX as interacting with CP, as determined by Shakeel et al. (1), are given in orange. *(bottom row)* Regions corresponding to the lowest 1% or the lowest 5% predicted interaction energies are depicted as dark and light green bars, respectively. Experimentally determined anti-CP aptamer matches are shown in gray, while the locations of stem loops identified via SELEX as interacting with CP are given in orange. **B)** Dependence of the interaction-energy BSC on z-score cutoff together with the corresponding p-values (black) for HPeV1 VP0, VP3 and VP1. The native BSC is given in red, while the median values of BSC for shuffled gRNA and CP sequences are given in blue.

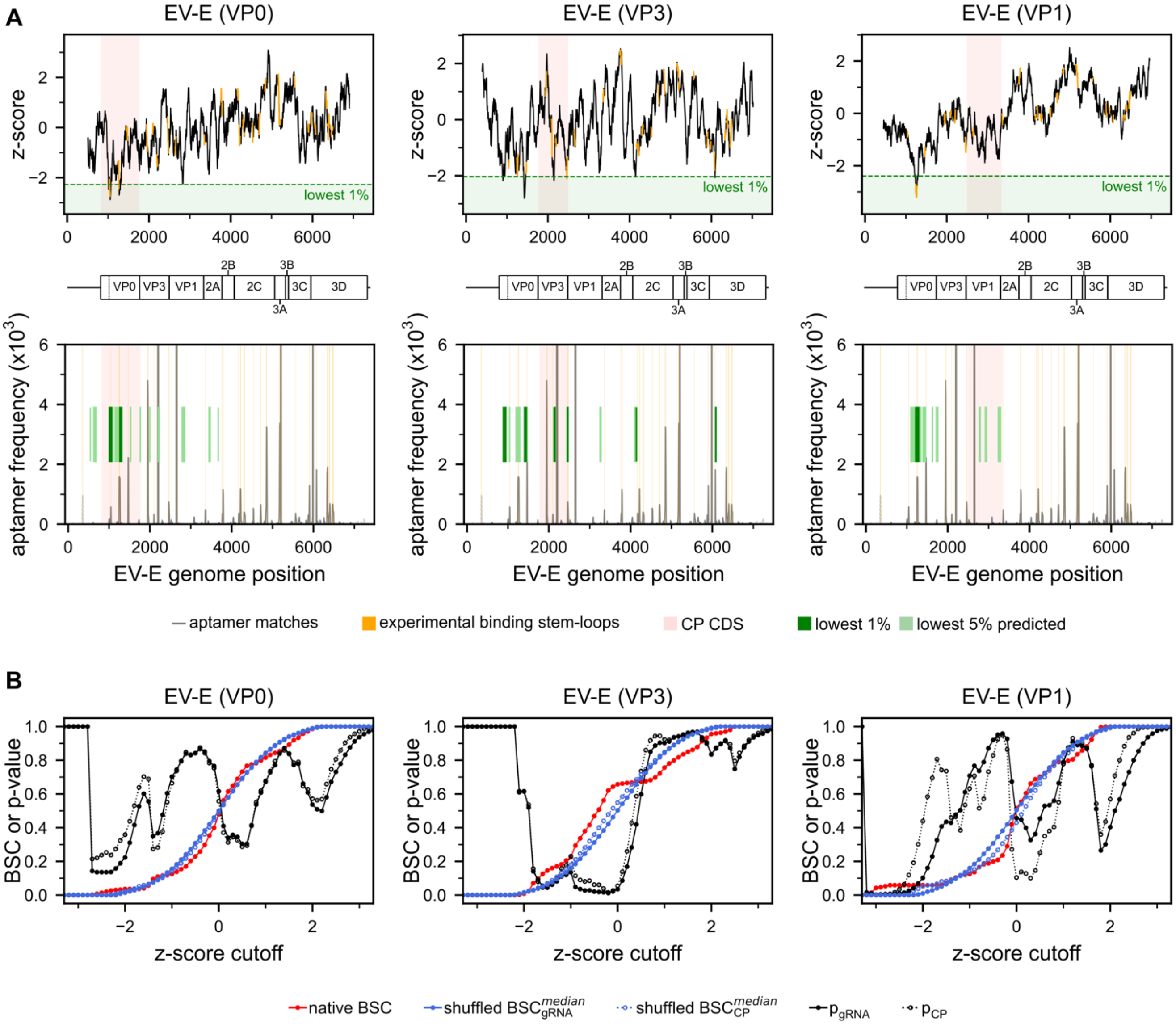

Figure SI6. Theoretical interaction energies of the EV-E capsid subunits along the genome and statistical analysis of BSC. A) *(top row)* Z-scores of the predicted relative interaction energies for VP0, VP3 and VP1 along the EV-E genome. The CDS of the corresponding CP subunit is highlighted in light red. Positions of the peaks above background in the anti-CP aptamer matches along the genome, as determined by Chandler-Bostock et al. (2), are depicted in orange. *(bottom row)* Regions corresponding to the lowest 1% or the lowest 5% predicted interaction energies are depicted as dark and light green bars, respectively. Experimentally determined anti-CP aptamer matches are shown in gray, while the peaks above background in the anti-CP aptamer matches are given in orange **B)** Dependence of the interaction-energy BSC on z-score cutoff together with the corresponding p-values (black) for EV-E VP0, VP3 and VP1. The native BSC is given in red, while the median values of BSC for shuffled gRNA and CP sequences are given in blue.

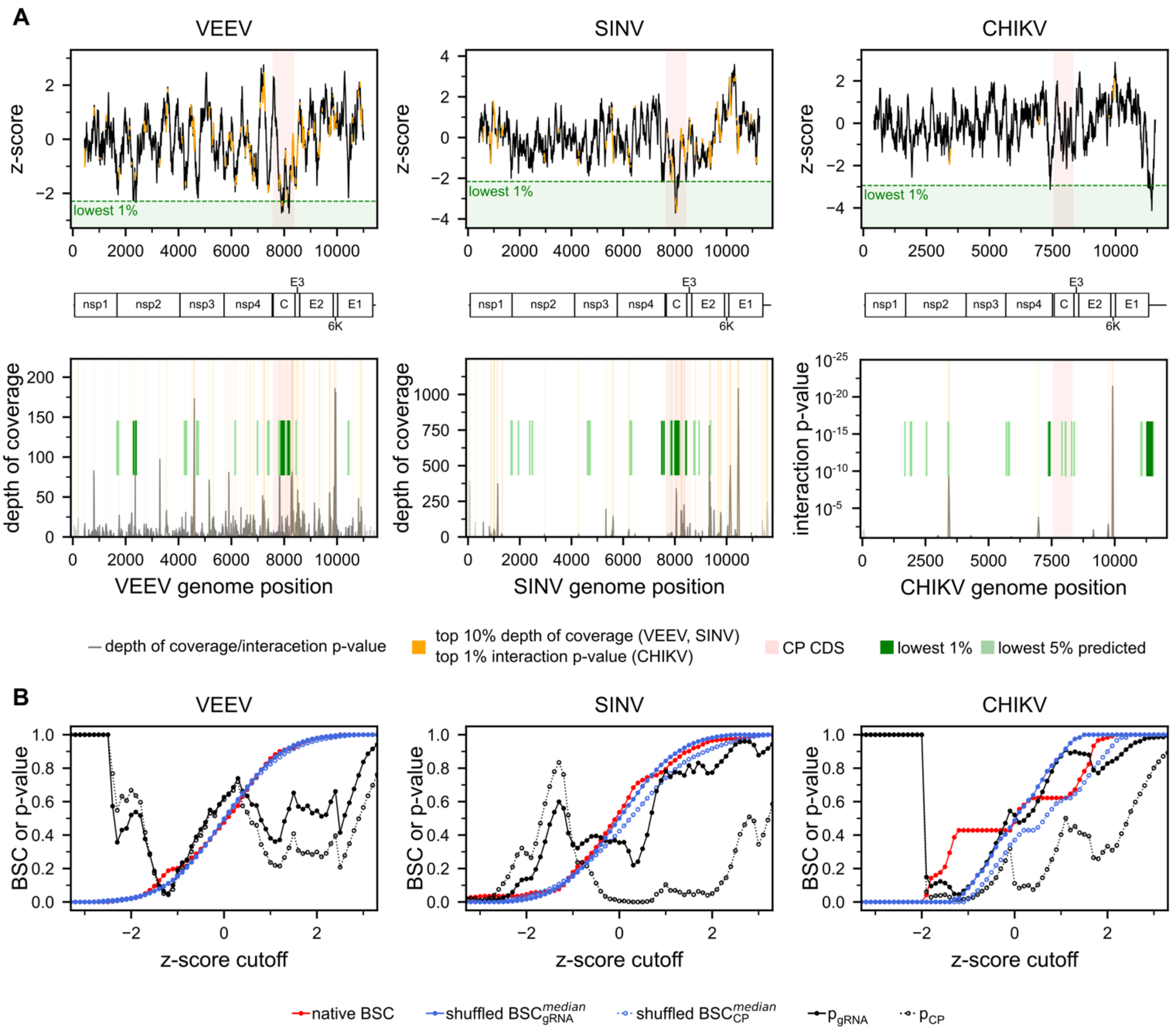

Figure SI7. Theoretical interaction energies of CP along the viral genome and comparison to CLIP-seq data for VEEV, SINV and CHIKV. A) *(top row)* Z-scores of the predicted relative interaction energies for CP along the VEEV, SINV and CHIKV genome. Location of the CP CDS is highlighted in light red. Regions belonging to the top 10% in read coverage are depicted in orange for VEEV and SINV (3, 4), while regions in the top 1% interaction p-values are depicted in orange for CHIKV (5). *(bottom row)* Regions corresponding to the lowest 1% or the lowest 5% predicted interaction energy are depicted as dark and light green bars, respectively. Depth of coverage/interaction p-value is shown in gray. **B)** Dependence of the interaction-energy BSC on z-score cutoff together with the corresponding p-values (black). The native BSC is given in red, while the median values of BSC for shuffled gRNA and CP sequences are given in blue.

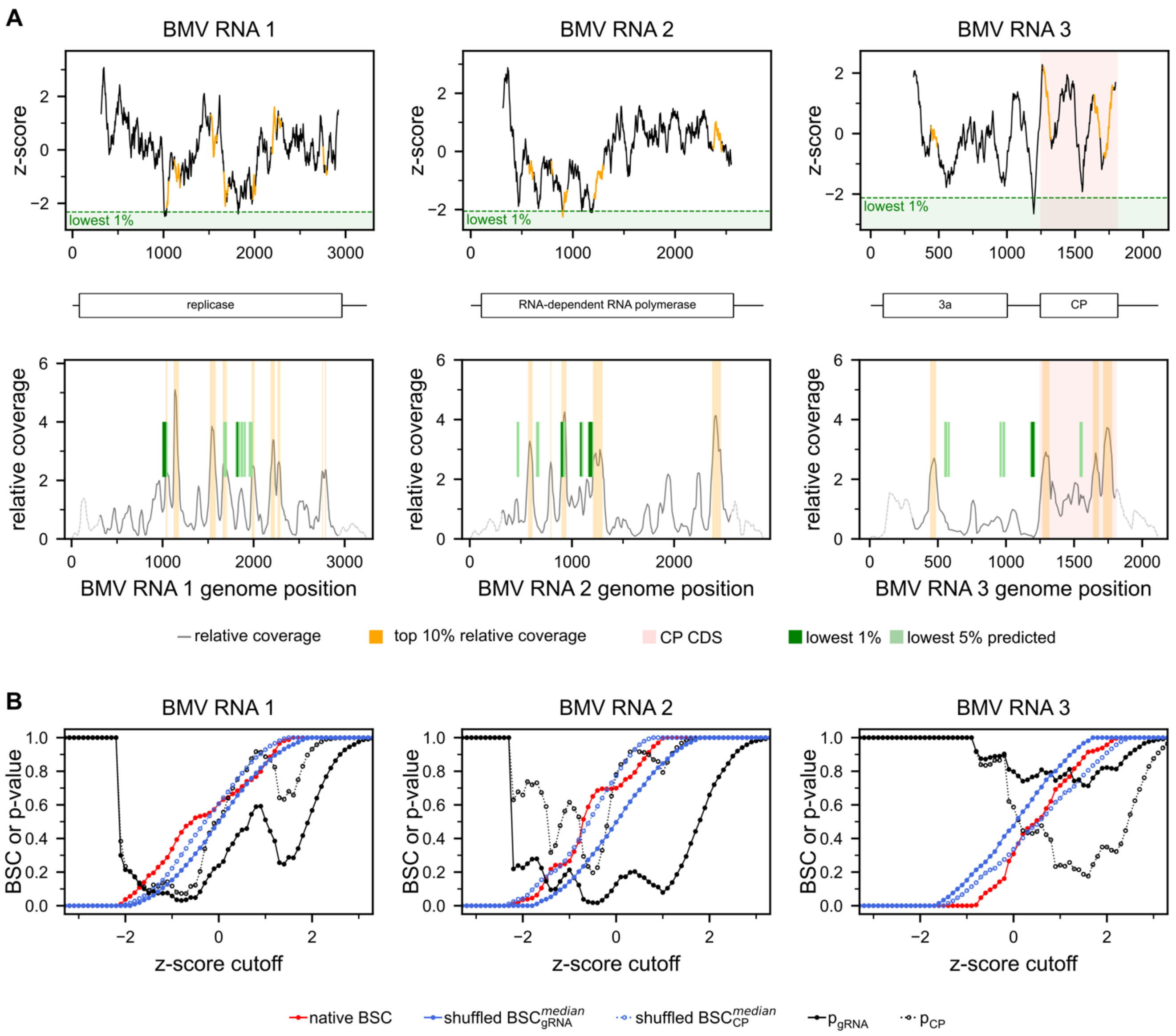

Figure SI8. Theoretical interaction energies of CP along the BMV genome and comparison to CLIP-seq data. A) *(top row)* Z-scores of the predicted relative interaction energy for CP along BMV RNA1, RNA2 and RNA3. The location of the CP CDS is highlighted in light red. Regions belonging to the top 10% in read coverage are depicted in orange (6). *(bottom row)* Regions corresponding to the lowest 1% or the lowest 5% predicted interaction energies are depicted as dark and light green bars, respectively. Relative coverage is shown in gray, while regions corresponding to the top 10% relative coverage are given in orange. **B)** Dependence of the interaction-energy BSC on z-score cutoff together with the corresponding p-values (black). The native BSC is given in red, while the median values of BSC for shuffled BMV gRNA and CP sequences are given in blue.

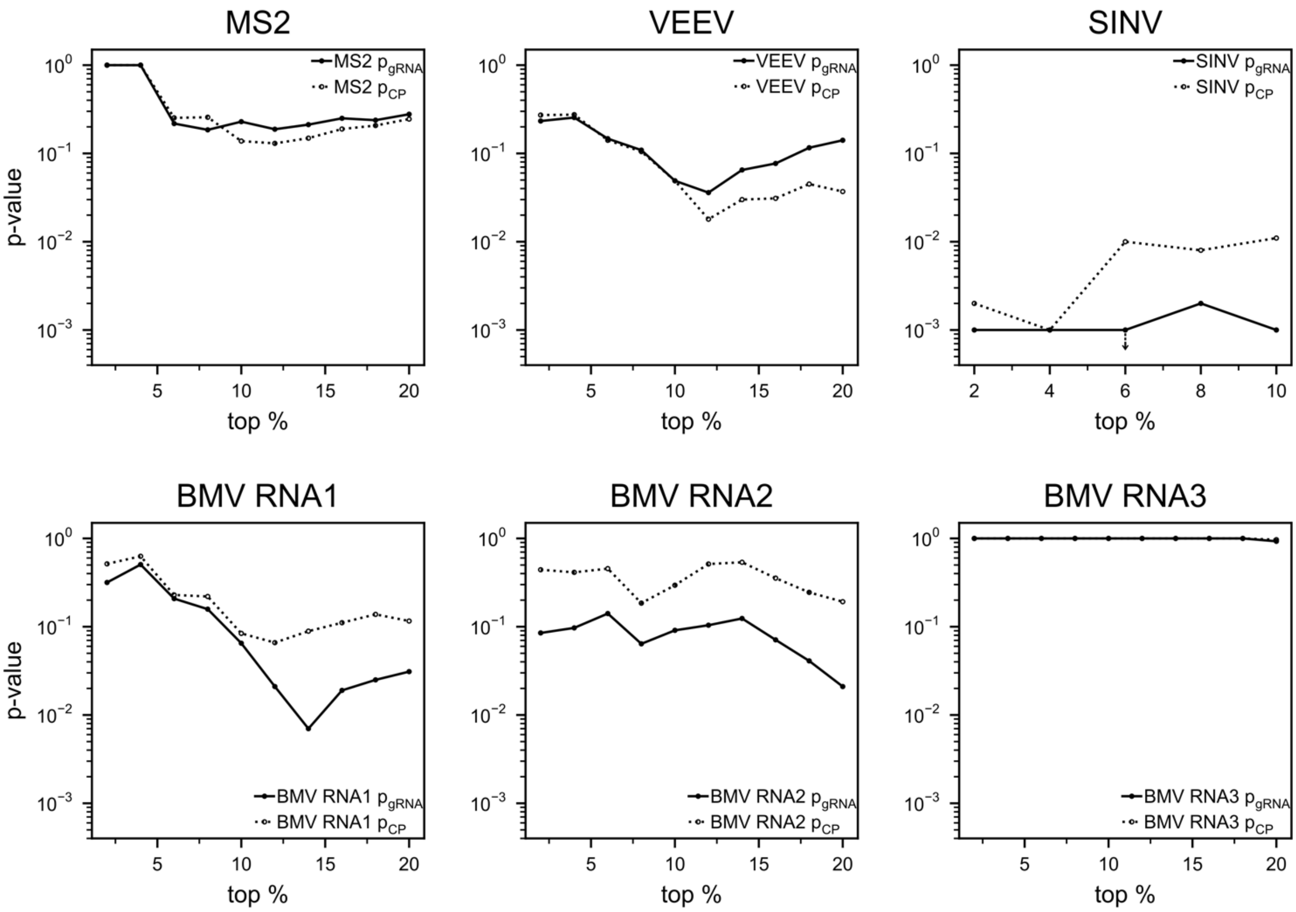

Figure SI9. Analysis of different cutoffs used to define the reference binding sites in CLIP experiments. Reference binding sites were defined by the gRNA positions that were among the best according to read coverage, with different cutoffs explored. For MS2 (7), VEEV (3) and BMV (6), cutoffs between 2% and 20% were explored in steps of 2%. For SINV, the range was limited to 10%, as only a few data points above 0 were reported in the original study, making the exploration of high cutoffs obsolete (4). Similarly, since the number of data points above the baseline for CHIKV did not greatly exceed 1% of the gRNA (5), we have limited our analysis to this single cutoff in that case (Figure SI7, right). For each of the analyzed viruses, the lowest obtained p-value for the interaction-energy BSC for z-score cutoffs $\leq$ -1 is shown as a function of the cutoff used to define the reference binding sites. Solid lines indicate p-values obtained for shuffled gRNA sequences, while dashed lines indicate p-values obtained for shuffled CP sequences. The downward arrow indicates a p-value < 10^{-3}.

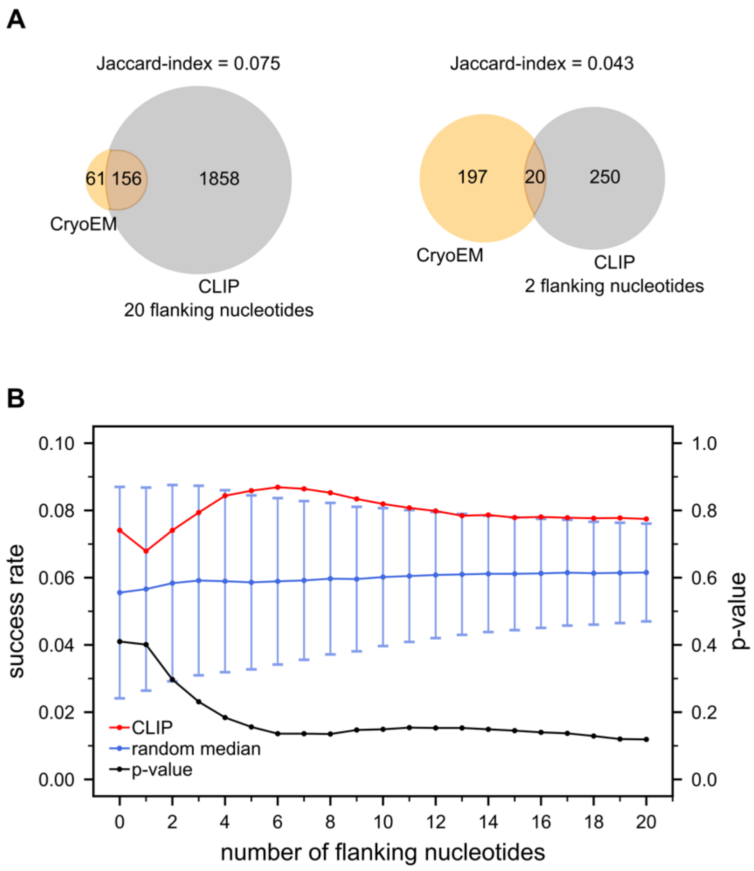

Figure SI10. Comparison of MS2 gRNA/CP binding site detected by cryo-EM and CLIP. A) *(left)* Overlap between the set of gRNA nucleotides in the binding stem loops seen in the MS2 cryo-EM structure (yellow) with the set of nucleotides in the CLIP-detected binding regions reported by Rolfsson et al. (7) (grey). The CLIP regions were determined by including the read-coverage peaks and 20 flanking residues to the left and 20 to the right of them; *(right)* same as on the left, but now with the CLIP-detected binding regions including the read-coverage peaks and just 2 flanking nucleotides to the left and 2 to the right of them. In this way, the number of binding site positions is approximately the same for cryo-EM and CLIP. The Jaccard indices are given above the Venn diagrams. **B)** Quantification of the degree to which CLIP-detected binding regions in MS2 match the cryo-EM-detected binding regions. The red curve captures the fraction of nucleotides in the cryo-EM-detected binding stem loops which were also detected in the CLIP experiment, as a function of the number of flanking residues surrounding the read-coverage peaks that were included to define the CLIP binding regions. The random sample was determined by placing 1000 times the same number of equally sized stretches on the gRNA at random. The median success rate among these randomized trials in predicting cryo-EM positions is given in blue with error bars representing the standard deviation. The reported p-values correspond to the fraction of such randomized trials in which the overlap with cryo-EM was equal or better than that in the real CLIP experiment.

Species	Method	Protein	IS in CDS			Interaction energy	
			In top 10%	In top 5%	In top 1%	shuffled$_{gRNA}$	shuffled$_{CP}$
BMV RNA1	CP	CLIP-seq	-	-	-	*	*
BMV RNA2	CP	CLIP-seq	-	-	-	*	-
BMV RNA3	CP	CLIP-seq	Yes	Yes	Yes	-	-
SFV	CP	PAR-CLIP	Yes	No	No	-	-
VEEV	CP	CLIP-seq	Yes	Yes	Yes	*	**
SINV	CP	CLIP-seq	Yes	Yes	No	***	**
CHIKV	CP	CLIP-seq	No	No	No	**	**
MS2	CP	CLIP-seq	Yes	No	No	-	-

* at least one z-score cutoff ≤ -1 with p-value <0.1

** at least one z-score cutoff ≤ -1 with p-value <0.05

*** at least one z-score cutoff ≤ -1 with p-value <0.01

Table SI1. Comparison between predicted gRNA/CP interaction sites and those observed by CLIP. Analyzed viruses and their CPs, together with the method used to study the gRNA/CP interaction. In the column labeled with “IS in CDS” (interaction site in the coding sequence), “yes” corresponds to the fact that there is at least one peak in the CP CDS that is among the top 10%, 5%, or 1% of sites in the entire gRNA when it comes to CLIP read coverage. Stars indicate significance for BSC for shuffled gRNA and shuffled CP sequences. In the case of VEEV (3), BMV (6), MS2 (7) and SINV (4), the top 10% of read coverage were used to define reference binding sites for comparison with interaction energies, while in the case of CHIKV the top 1% were used because of limited numbers of data points reported in the original study (5).

REFERENCES

1. Shakeel,S., Dykeman,E.C., White,S.J., Ora,A., Cockburn,J.J.B., Butcher,S.J., Stockley,P.G. and Twarock,R. (2017) Genomic RNA folding mediates assembly of human parechovirus. *Nat. Commun.*, **8**.

2. Chandler-Bostock,R., Mata,C.P., Bingham,R.J., Dykeman,E.C., Meng,B., Tuthill,T.J., Rowlands,D.J., Ranson,N.A., Twarock,R. and Stockley,P.G. (2020) Assembly of infectious enteroviruses depends on multiple, conserved genomic RNA-coat protein contacts. *PLoS Pathog.*, **16**, 1–23.

3. Carey,B.D., Akhrymuk,I., Dahal,B., Pinkham,C.L., Bracci,N., Finstuen-Magro,S., Lin,S.C., Lehman,C.W., Sokoloski,K.J. and Kehn-Hall,K. (2020) Protein Kinase C subtype δ interacts with Venezuelan equine encephalitis virus capsid protein and regulates viral RNA binding through modulation of capsid phosphorylation. *PLoS Pathog.*, **16**, 1–25.

4. Sokoloski,K.J., Nease,L.M., May,N.A., Gebhart,N.N., Jones,C.E., Morrison,T.E. and Hardy,R.W. (2017) Identification of Interactions between Sindbis Virus Capsid Protein and Cytoplasmic vRNA as Novel Virulence Determinants. *PLoS Pathog.*, **13**, 1–29.

5. Kiser,L.M., Sokoloski,K.J. and Hardy,R.W. (2021) Interactions between capsid and viral RNA regulate Chikungunya virus translation in a host-specific manner. *Virology*, **560**, 34–42.

6. Ni,P., Vaughan,R.C., Tragesser,B., Hoover,H. and Kao,C.C. (2014) The plant host can affect the encapsidation of brome mosaic virus (BMV) RNA: BMV virions are surprisingly heterogeneous. *J. Mol. Biol.*, **426**, 1061–1076.

7. Rolfsson,Ó., Middleton,S., Manfield,I.W., White,S.J., Fan,B., Vaughan,R., Ranson,N.A., Dykeman,E., Twarock,R., Ford,J., *et al.* (2016) Direct Evidence for Packaging Signal-Mediated Assembly of Bacteriophage MS2. *J. Mol. Biol.*, **428**, 431–448.
